# The volume and monetary value of human milk produced by the world's breastfeeding mothers: Results from a new tool

**DOI:** 10.3389/fpubh.2023.1152659

**Published:** 2023-03-30

**Authors:** Julie P. Smith, Alessandro Iellamo, Tuan T. Nguyen, Roger Mathisen

**Affiliations:** ^1^National Centre for Epidemiology and Population Health, College of Health and Medicine, The Australian National University, Canberra, ACT, Australia; ^2^Crawford School of Public Policy, College of Asia and the Pacific, The Australian National University, Canberra, ACT, Australia; ^3^Independent Researcher, London, United Kingdom; ^4^Alive and Thrive, FHI 360/FHI Solutions, Hanoi, Vietnam

**Keywords:** breastfeeding, economic evaluation, feminist economics, milk production, food systems, maternal and child health, reproductive labor, unpaid work and production

## Abstract

The Mothers' Milk Tool was developed to make more visible the economic value contributed to society by women's unpaid care work through breastfeeding infants and young children. This manuscript describes the development and display key features of the tool, and reports results for selected countries. For the development, we used five steps: (1) defining the tool by reviewing existing tools and scholarly literature to identify uses, approaches, design features, and required data characteristics for a suitable product; (2) specifying the best open-access data available for measurement and easy updating; (3) analyzing development options; (4) testing predictive models to fill identified breastfeeding data gaps; and (5) validating the tool with prospective users and against previous research. We developed an Excel-based tool that allows working offline, displaying preloaded data, imputing data, and inputting users' data. It calculates annual quantities of milk produced by breastfeeding women for children aged 0–35.9 months, and the quantities lost compared to a defined biologically feasible level. It supports calculations for an individual mother, for countries, and global level. Breastfeeding women globally produce around 35.6 billion liters of milk annually, but 38.2% is currently “lost” due to cultural barriers and structural impediments to breastfeeding. The tool can also attribute a monetary value to the production. In conclusion, the Mothers' Milk Tool shows what is at risk economically if women's important capacity for breastfeeding is not protected, promoted, and supported by effective national policies, programs, and investments. The tool is of value to food and health policymakers, public officials, advocates, researchers, national accountants and statisticians, and individual mother/baby dyads, and will assist consideration of breastfeeding in food balance sheets and economic production statistics. The tool supports the 2015 Call to Action by the Global Breastfeeding Collective by facilitating the tracking of progress on breastfeeding targets.

## Highlights

- Milk provided by breastfeeding mothers is a crucial but largely invisible national food resource.
- The economic value of women's milk production can and should be measured, to ensure this contribution is visible and properly valued.
- Much valuable production of this food is “lost” due to cultural barriers and structural impediments to breastfeeding.
- Supportive breastfeeding culture is an important national capital asset with large economic value.
- Breastfeeding provides food security for a country's children while minimizing food system pressures on the environment.

## 1. Introduction

### 1.1. The importance of breastfeeding for nutrition and health

A large volume of epidemiological evidence and many studies reaffirm the nutrition and health impacts of breastfeeding and support a growing global focus on the investment case for breastfeeding promotion. Lack of breastfeeding costs lives, and deprives young children, their mothers, and their countries of important health, human capital, and economic impacts (1–3).

The economic contribution made by women through breastfeeding is still largely invisible in economic data and fiscal decision-making (3). Applying economic frameworks for analyzing human milk production may raise awareness of the public policy importance of women's economic productivity in this unique unpaid care work.

Economists have long been aware of the limitations of conventional economic accounting systems for measuring economic activity and material well-being (4–8). Feminist economists have criticized the failure of the System of National Accounting (SNA) to count women's unpaid and reproductive work as economic production and its exclusion from supposedly objective measures such as Gross Domestic Product (GDP), which, in principle, covers all transactions in economic goods and services. In her 1988 book, *Counting for Nothing*, Marilyn Waring discussed (9) the need to value women's work, including reproductive and care work such as breastfeeding, in GDP.

Economic statisticians and national accounting experts have now acknowledged the crucial, unpaid role of families in building human capital, such as through investments of parental time in health care and education (10). Indeed, a 2009 review of GDP measurement for the French President led by two of the world's leading economists, Nobel prize-winners Amartya Sen and Joseph Stiglitz (11), cited human milk production as an example of how current practices for measuring GDP devalued women's unpaid work and biased policymaking. They stated that breastmilk constitutes a “serious omission in the valuation of home-produced goods, which is clearly within the SNA production boundary, is quantitatively non-trivial and has important implications for public policy and child and maternal health.”

The invisibility of women's economic contribution in national economic statistics contributes to policy bias against protecting and resourcing nonmarket production (12). Scholars have pointed out the significant consequences of the lack of recognition of women's unpaid work for policy advocacy, design, implementation, and evaluation (12–14). Policies that acknowledge the importance of the valuable non-market production involved in breastfeeding, and the need to protect it, include “breastfeeding-friendly” health and maternity care services, more adequate paid maternity leave, and effective regulation of marketing and promotion of breastmilk substitutes. Such policies are identified in the WHO/UNICEF *Global Strategy* (15), and more recently represented in the 2015 Call for Action on Breastfeeding (16). The latter particularly emphasized the importance of strengthening monitoring systems to track progress toward achieving global and national policy targets on breastfeeding.

Ignoring breastfeeding also discounts the highly valuable role families, and in particular, mothers, play in human capital development (10). However, more than three decades on from changes to the SNA in 1993 that allow for counting human milk production in GDP, the problem of valuing breastfeeding in economic statistics remains largely unaddressed and ignored in public policy formulation (17).

### 1.2. Including human milk in food statistics and GDP

Broadly, there are three types of macroeconomic studies of breastfeeding, including studies on (1) the economic and health system costs of low breastfeeding rates; (2) the costs of breastfeeding protection, promotion, and support programs; and (3) the economic value of breastfeeding and economic costs of ‘lost milk'.

Two existing online tools - the Cost of Not Breastfeeding (CNB) Tool, and the World Breastfeeding Costing Initiative (the WBCi Costing Tool) - provide the means to calculate the country-level costs of not breastfeeding (2, 18), and the financing needs to invest in implementing strategies on infant and young child feeding (19). The PROFILES Tool for Calculating Health, Child Spacing and Economic Benefits of Breastfeeding (BOB) was developed as part of a larger process of nutrition policy dialogue to calculate the costs of not breastfeeding alongside the macroeconomic value of breastfeeding (18, 20) but has not been widely used or promoted.

The Mothers' Milk Tool has been developed to complement and build on these other tools. The tool makes visible the economic value contributed to society by women's unpaid care work through breastfeeding infants and young children.

### 1.3. Aims

To develop an online and downloadable tool to estimate the economic value of breastfeeding and the monetary value of “mothers' milk.” We envisage that this evidence-based and user-friendly “mothers' milk” tool will be used by policymakers, advocates, national accountants/statisticians, and researchers to estimate the economic value of breastfeeding and the economic costs of “lost mothers' milk” to support advocacy for breastfeeding-friendly environments. Specific objectives are to (1) describe the development process of the tool, (2) display key features of the tool, and (3) report estimates for selected countries.

## 2. Methods

The design of the Mothers' Milk Tool draws on more than 40 years of research. The development process used 5 steps: define, measure, analyze, design and develop, and verify - DMADV (21, 22).

### 2.1. Step 1: Definition

#### 2.1.1. Users and uses

In the first step, existing tools and scholarly literature estimating the economic value of breastfeeding were reviewed to identify uses, approaches, design features, and data that could be adopted in the development and definition of a suitable product. To identify the key design and methodological issues for such studies, a detailed review was conducted to identify all relevant studies of the macroeconomic value of breastfeeding, and extract summary data on their coverage, data, methods, and results.

The review identified that significant but diverse literature exists on the economic value of breastfeeding. The review found around 65 country estimates of the macroeconomic value of breastfeeding, for a total of around 25 countries.

The geographic areas covered included Europe, Asia, America, Africa, and Australasia. Several studies produced estimates for groups of countries, and/or for the whole world. Estimates go as far back as 1908, and up to 2018, and for several countries in the 1950s, 1960s, and 1970s. Most identified the quantities and values of milk produced for infant and young child populations aged 0–23.9 months. However, some country estimates were for ages 0–35.9 months. A small number of estimates were of breastmilk supplied for infants only, aged 0–11.9 months or less. The results of the review confirmed not only the relevance but also the feasibility, utility, and sustainability of counting breastmilk as part of national economic statistics. Norway's reporting systems were identified as a model for initial steps toward making the value of mothers' milk visible within a food surveillance framework.

This review also considered the potential uses and users of the tool. Most studies aimed to improve the visibility of breastfeeding; motivations included the desire to provide better scientific information for public policy and budgeting decisions; reduce the public invisibility of women's productivity, including breastfeeding; and highlight the need for measures to prevent or address declines in breastfeeding. Some studies were conducted by nutritionists working for international agencies, while others advocated for the government to develop breastfeeding policies and programs. For example, in the early 1970s, World Bank nutrition advisor Alan Berg documented the expanding economic loss associated with formula feeding replacing breastfeeding in countries such as Chile, Kenya, Singapore, and the Philippines over the previous decade, aiming to motivate public action to reverse this decline (23). Likewise, pediatrician Jon Rohde (24–26) calculated the quantities of human milk production in Indonesia during the 1970s and 1980s to emphasize the importance of breastfeeding in the second year of that country's food supply and nutrition policies. A study led by nutritionist Stina Almroth in 1979 presented estimates of the economic value of breastfeeding for Ghana and the Ivory Coast to inform FAO considerations of breastfeeding as infant food, for infant health protection, and child spacing (27).

Later studies from the 1990s demonstrated the magnitude of production and the macroeconomic value of mother's milk for countries in Latin America, Sub-Saharan Africa, China, and India (27–32). Studies led by (25, 26) pediatrician Arun Gupta produced estimates for India (28, 32). The PROFILES project (see above) provided estimates of breastmilk production and its financial value for Bolivia, China, and the countries of West Francophone Africa. This showed for example that the volume of human milk produced in China was around 4 billion liters in 2001 (20, 33). Notably, at a time when human milk was priced at around $50 a liter in high-income countries such as the US and Norway, the 1997 study of the countries of Sub-Saharan Africa a study by nutritionists Anne Hatloy and Arne Oshaug found that given a monetary value of just $1 per liter, the economic value of human milk production ranged from 5 to 15% of the GDP of those countries (34). Until 1994 (35), nearly all studies calculated the value of human milk by estimating output in physical units and then valuing it using the market price of an alternative commodity.

#### 2.1.2. Required tool outputs and other capabilities

The review of studies indicated that measures of actual, potential, and lost milk were the common outputs of interest to users. Also, useful would-be comparisons with national or international targets and benchmarks as well as the capacity to calculate results for significant age categories within the 0–35.9 months age range. For example, some studies examined 12–23.9 months, or 0–5.9 months, while the majority looked at 0–23.9 months including 0–11.9 months.

This analysis of the literature also indicated that the tool should have both online and offline versions to cater to diverse uses as well as the intended end use. Potential use includes calculating the production of human milk within food surveillance systems, allowing policymakers to use the results to monitor the results of food security and nutrition policies. Another potential use is the provision of evidence for non-government advocacy, where users from civil society or international agencies could demonstrate the economic significance of breastfeeding and highlight the need for policies targeting breastfeeding protection, promotion, and support.

The review also demonstrated that the tool must present key results for selected countries as well as the world, allowing users to see country-level results from a wider comparative perspective. The design also needed to be flexible to meet the main customization needs of policymakers, advocates, researchers, and individuals worldwide, and to allow for future updates and enhancements.

In light of the available budget for tool development, a basic version was planned for rapid development and release, to add further enhancements over time based on feedback from users. The type of enhancements being considered is further discussed in the concluding section.

### 2.2. Step 2: Measurement

Step 2 specified the best open access data available for measurement, and assessed which data allowed future modules to be easily updated. Previous studies used a variety of data sources for key inputs to the calculations, making comparisons difficult. This highlighted the need for the tool to use consistently available open-access data for countries to make the best estimates. It is also important for future modules to be easily or automatically updated with key default data in a timely and efficient manner.

There are four key measures. First, the number of infants and young children aged 0–35.9 months is approximated by UNICEF databases (36). UN population estimates data on live births for the base year and estimated number of children in the first, second, and third years of life. Second, we used country survey data on continued breastfeeding rates, such as from Demographic and Health Surveys (DHS) and Multiple Indicator Cluster Surveys (MICS), as the basis for predicting breastfeeding rates for infants and young children by month, 0–35.9 months for most countries (37). Third, estimates of human milk intake by child age (i.e., by month, every six months, and overall three years), based on reliable and commonly used studies of energy intake in breastfed children. This is a fixed element of the tool and provides for a total of 431 liters of milk produced over the 36 months of lactation, derived from two authoritative studies, and based on their published estimates for partially breastfed infants, converted from grams to milliliters (34, 38). Fourth, a price per liter of human milk of US$100 per liter is based on the official price for fresh human milk within Norway's human milk banking system (39, 40). Alternative prices are summarized and discussed briefly in Supplementary material 2.

### 2.3. Step 3: Analysis of tool design options

Due to the different potential uses and users and the limitations of the data, we considered two main stages for the tool development: a basic module and a customizable module.

The basic module would include a dashboard that shows the findings and estimations of a selected country or the world using the newest possible preloaded data. This module can also impute missing values of continued breastfeeding to provide a more precise estimate of the value of breastmilk.

The tool would also provide for customization, so users can input alternative data such as breastfeeding rates, the size of the population, the market value of human milk, and the currency exchange rate for the country of interest.

The tool would allow an individual mother to enter her own breastfeeding experience to calculate the amounts of milk provided for her child.

### 2.4. Step 4: Tool development

Tool development focused on identifying and pre-loading key data sources and developing a suitable predictive model for breastfeeding rates.

It also required the investigation of a suitable basis for estimating milk production levels and exploring sources of evidence on the daily milk intake of breastfeeding children. A further area of investigation was the biologically feasible potential production. The difference between this and actual production levels is the “lost” milk production calculated by the tool.

The key data sources and analyses behind the estimates are discussed in Supplementary material 1.

#### 2.4.1. Initial development

Initial investigation of the goals for the tool identified the need for a downloadable tool that can be easily updated with low investment. This stage also identified the need for the user to be provided with key parameters which were fixed in the tool, as well as the potential for the user to make calculations using alternative data sources on breastfeeding or numbers of children born and breastfed.

While the main interest was in country-level estimates, sub-national and individual mother calculations were also identified as useful for meeting tool goals.

The primary goal identified was advocacy, but additional potential uses included mothers calculating production volume or values over the breastfeeding period as motivation, as well as health professionals supporting and encouraging breastfeeding mothers.

#### 2.4.2. Internal discussions, external consultation, and improvements

Discussions held fortnightly during 2021 by members of the Organization 1 and Organization 2 teams resulted in agreed-upon priorities for the first stage basic version of the tool, and priorities for enhancement in future upgrades.

The most important revisions during the development phase were to align the tool with the 0–35.9 age group for infant and young child feeding. Many previous studies were for 0–11.9 months, or at most 0–23.9 months. The tool is unique in its provision of data for the extended age range, which fits into the WHO/UNICEF recommendations for breastfeeding beyond 2 years of age.

The development of the tool also considered the maximum biologically feasible levels of breastfeeding. The tool calculates the lost milk on the basis that 98% of mothers can breastfeed, based on contemporary data from Norway (41) and a review of the median weaning age in traditional or non-industrial populations (42).

Data gaps also influenced tool design. Although DHS surveys include breastfeeding data for 0–35.9 months, the MICS did not. Also, few high-income countries consistently collect data, especially beyond 11.9 months, and some had no recent data. Many did not have data on exclusive breastfeeding. With the substantial data gaps evident during the analysis phase, it became necessary to invest in developing a prediction model for monthly breastfeeding rates for children ages 0–35.9 months. The tool bridges these data gaps to calculate predictions of breastfeeding at each month of age from available national data. This predictor uses a cubic regression model for most countries, though in some countries the best fit was predicted by a linear regression model.

In several countries where traditional breastfeeding practices are largely intact, the value of breastfeeding was substantial to the market economy (as measured by GDP). This pointed to the importance of deciding on how to attribute a monetary value to breastfeeding to allow this comparison with the conventional official measurement of economic value. Few studies attempted to value the act of breastfeeding *per se* (43, 44); while breastfeeding can be conceived of as an unpaid care service within an economic accounting framework, most studies calculated its value by reference to a price proxy for the human milk produced; that is, as a food commodity. The focus was on measuring the economic value of breastfeeding by estimating the national monetary cost of buying or importing commercial milk formula. Nearly all studies used the cost of replacing breastmilk with either fresh or formula milk to infer the economic value of breastfeeding.

The Mothers' Milk Tool places a monetary value of US$100 on human milk produced by breastfeeding women. This is linked to the price of fresh human milk exchanged within the not-for-profit hospital milk bank network in Norway where a US$20 per liter reimbursement of costs is made to donating mothers (39). Price increases are regulated by the Norwegian government and increases reflect cost recovery principles since the 1990s. Alternative prices were evaluated in previous studies (43–45) (see Supplementary material 2).

There were two stages of piloting the Mothers' Milk Tool, which occurred during the early weeks of 2022. We aimed for a range of potential user groups to be represented in the two testing groups, coming from a diverse range of countries and global regions. Several improvements were implemented after piloting, mainly to improve presentation and clarity and address functionality issues.

#### 2.4.3. Formatting the tool

A suggestion from reviewers on the first version was to follow a branding guideline. The branding guidelines from Organization 2 were selected and used consistently to design the Mothers' Milk Tool. Based on the comments, additional information was added, such as the introduction, policy brief, and references, to make the tool more comprehensive and standalone.

#### 2.4.4. Description and display of key features of the tool

**Figure 1:**
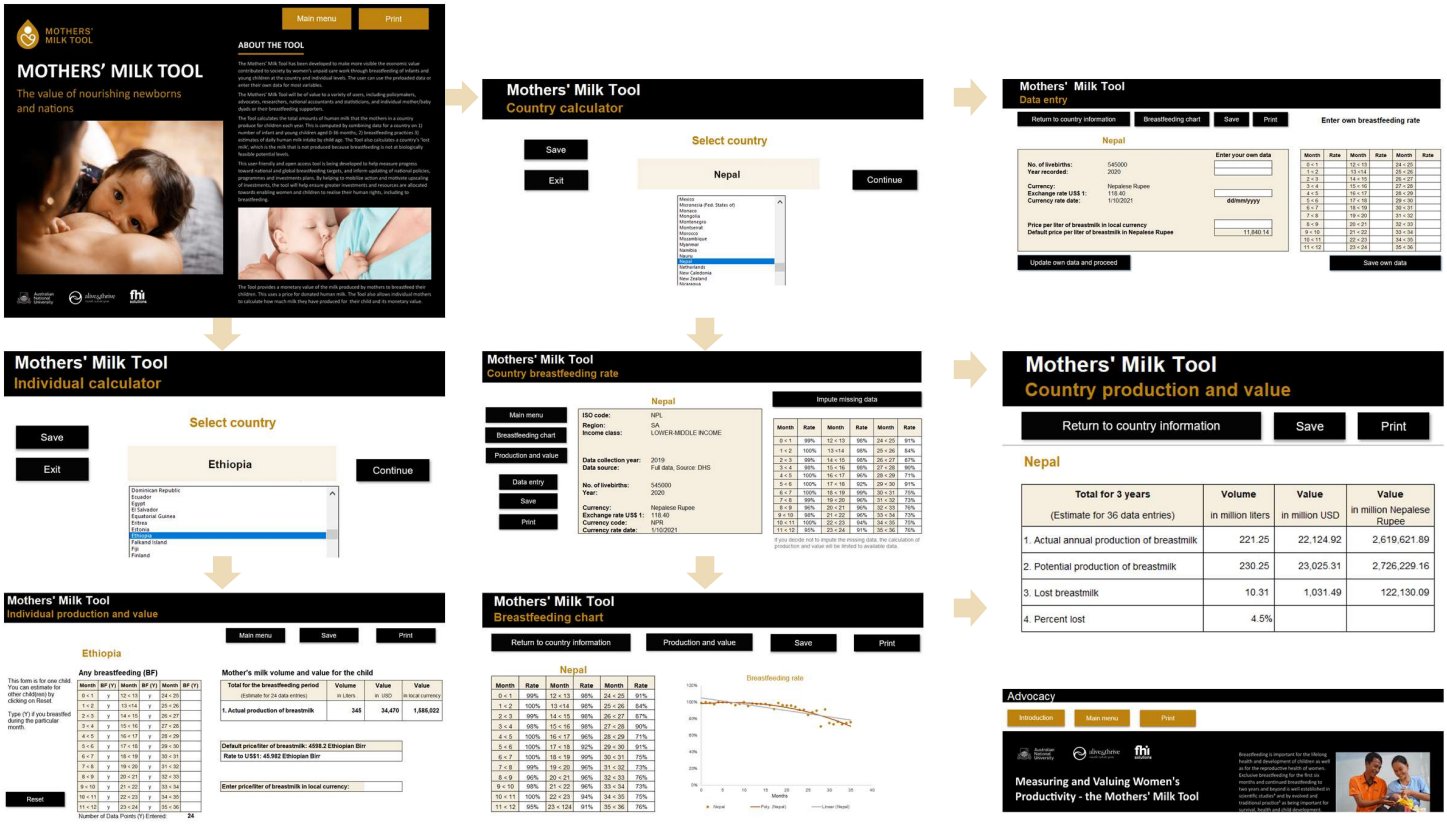
Key functions of the Mothers' Milk Tool. Authors created this figure using snapshots of the Mothers' Milk Tool offline (https://mothersmilktool.org). The human identifiable images are licensed for personal, business or commercial purposes.

Figure 1 illustrates the key functions of the Mothers' Milk Tool, including both country and individual calculators. The individual calculator allows for the estimation of individual production and value for each child of a user based on the duration of breastfeeding. The user is to enter the information on the months she breastfed her child, and the tool will help to estimate the volume and value of breastmilk. The user can enter and obtain information for other children. This function could be used by breastfeeding counselors during breastfeeding promotion and support.

For the country calculator, the Mothers' Milk Tool will provide the country's breastfeeding rates and chart using preloaded data. When the data are not up-to-date or missing, the users have the option to input the missing data using the predictor or enter their own-source data. Using the data, the Mothers' Milk Tool will estimate the annual production, potential production, and lost breastmilk and their values.

Table 1 illustrates calculations for a single country for a single year for infants and young children aged <36 months.

**Table 1:** Estimate of production of human milk.

**Child age (months)**	**Proportion of children breastfed (%)**	**Number of children breastfed per month**	**Average volume of breastmilk consumed a day per child (L)**	**Estimated volume of breastmilk consumed a month per child (L)**	**Total actual annual production of breastmilk (million L)**
0 (<1)	93	55,800	0.59	18	0.99
1	89	53,400	0.68	20	1.08
2	85	51,000	0.71	21	1.08
3	82	49,200	0.68	20	1.01
4	79	47,400	0.69	21	0.98
5	78	46,800	0.59	18	0.83
6	72	43,200	0.55	17	0.71
7	68	40,800	0.4	12	0.49
8	63	37,800	0.48	14	0.55
9	58	34,800	0.67	20	0.70
10	51	30,600	0.5	15	0.46
11	48	28,800	0.48	14	0.42
12	34	20,400	0.37	11	0.23
13	29	17,400	0.37	11	0.19
14	24	14,400	0.37	11	0.16
15	21	12,600	0.37	11	0.14
16	20	12,000	0.37	11	0.13
17	16	9,600	0.37	11	0.11
18	14	8,400	0.37	11	0.09
19	12	7,200	0.37	11	0.08
20	11	6,600	0.37	11	0.07
21	10	6,000	0.37	11	0.07
22	9	5,400	0.37	11	0.06
23	8	4,800	0.37	11	0.05
24	0	–	0.24	7	–
25	0	–	0.24	7	–
26	0	–	0.24	7	–
27	0	–	0.24	7	–
28	0	–	0.24	7	–
29	0	–	0.24	7	–
30	0	–	0.24	7	–
31	0	–	0.24	7	–
32	0	–	0.24	7	–
33	0	–	0.24	7	–
34	0	–	0.24	7	–
35	0	–	0.24	7	–

Methodology based on Norwegian Health Directorate 2020 (46) and Smith et al. 2022 (47).

Table 2 summarizes yields that were assumed in previous studies.

**Table 2:** Average volume (liters) of human milk intake by a child and by month of age in studies on economic value of breastfeeding.

	**Months of infant age** ^*^		
Authors/Months of infant age^*^	**0–11.9**	**0–23.9**	**0–35.9**
Smith (48)	228	307	**–**
Norwegian Health Directorate (49)	225	306	**–**
WHO^*^ (38)	291 (214)	**–**	**–**
Aguayo et al.^*^ (29)	243 (225)	443 (436)	536 (518)
Smith (44)	224	331	**–**
WHO^*^ (50)	256 (226)	450 (421)	**–**
Hatloy and Oshaug (34)	230	369	462
National Nutrition Council (51)	228	307	**–**
Oshaug and Botten (35)	224	331	**–**
Gupta and Khanna (32)	201	347	**–**
Almroth et al. (27)	234	380	**–**
Rohde (25)	180	288	360
Berg (23)	247	375	**–**

* Values in brackets are for partial breastfeeding.

Table 3 provides information on sources of data on births and breastfeeding survey dates used in the calculations.

**Table 3:** Source of breastfeeding data.

**Country/Location**	**Source of breastfeeding data**	**Year**	**Annual Livebirths**
Australia	Australian infant feeding survey	2010	339,000
Brazil	Health and nutrition survey	2019	2,871,000
Canada	Community health survey	2009	402,000
India	National health family survey	2005–2006	24,143,000
Indonesia	Demographic and Health Survey	2017	4,466,000
Ireland	Breastfeeding on the Island of Ireland, Report 3	2013	57,000
Kenya	Demographic and Health Survey	2014	1,418,000
Nepal	Multiple Indicator Cluster Survey	2019	545,000
Nigeria	Demographic and Health Survey	2018	7,894,000
Norway	Directorate for Health and Social Affairs,	2020	60,000
Philippines	Demographic and Health Survey	2017	1,955,000
United Kingdom	National survey	2011	744,000
USA	National immunization survey	2018	3,991,000
Viet Nam	Multiple Indicator Cluster Survey	2013–2014	1,592,000
Global	UNICEF infant and young child feeding database	2020	136,077,713

### 2.5. Step 5: Tool validation

During development, data from several countries were entered into the tool, and results were compared with results from published studies for the relevant country to assess the validity of tool outputs (Supplementary material 3). This table compares results from the original study, with calculations using the tool. The calculations using the tool use the same birth and breastfeeding data as the original studies, but not the milk intakes/yields assumed in those studies, so differences arise mainly from differences in methodologies or differences in assumed yields. Reasons for variance are indicated in the table on this basis.

The tool was also validated by inviting country IYCF and breastfeeding experts to provide feedback on its functioning, usefulness, plausibility, and reliability of the results and underlying assumptions for that country. A total of 16 potential users responded to the invitation for testing the tool. Respondents were from 12 countries, and their self-described occupations or interest in the tool included advocate, nutritionist, economist, director, peer counselor, nutrition specialist, lactation consultant, medical doctor, and independent consultant. Feedback was centered on the functionality and utility of the tool. User feedback from testing is reported in Supplementary material 4.

#### 2.5.1. Country selection and estimates

Estimates were made for a selection of high-, middle- and low-income countries from the global regions, using the prediction model for all those countries where complete breastfeeding data was not available. These countries reflect a diversity of breastfeeding prevalence, some maintaining intact breastfeeding practices at levels consistent with those reported for non-industrial or historical populations, and others with very disrupted breastfeeding practices. Global production was estimated for low- and middle-income countries (LMICs) only due to data limitations for high-income countries (HICs).

For a small number of countries, the estimates were tested using historical data series, and for other countries, it was possible to compare the results of the tool with published estimates made at another point in time for the same country. The country selection also reflected large, medium, and small populations, which may approximate the extent to which they are a profitable market for the expansion of commercial milk formula and other baby food sales.

#### 2.5.2. Continuous tool improvement

**Figure 2:**
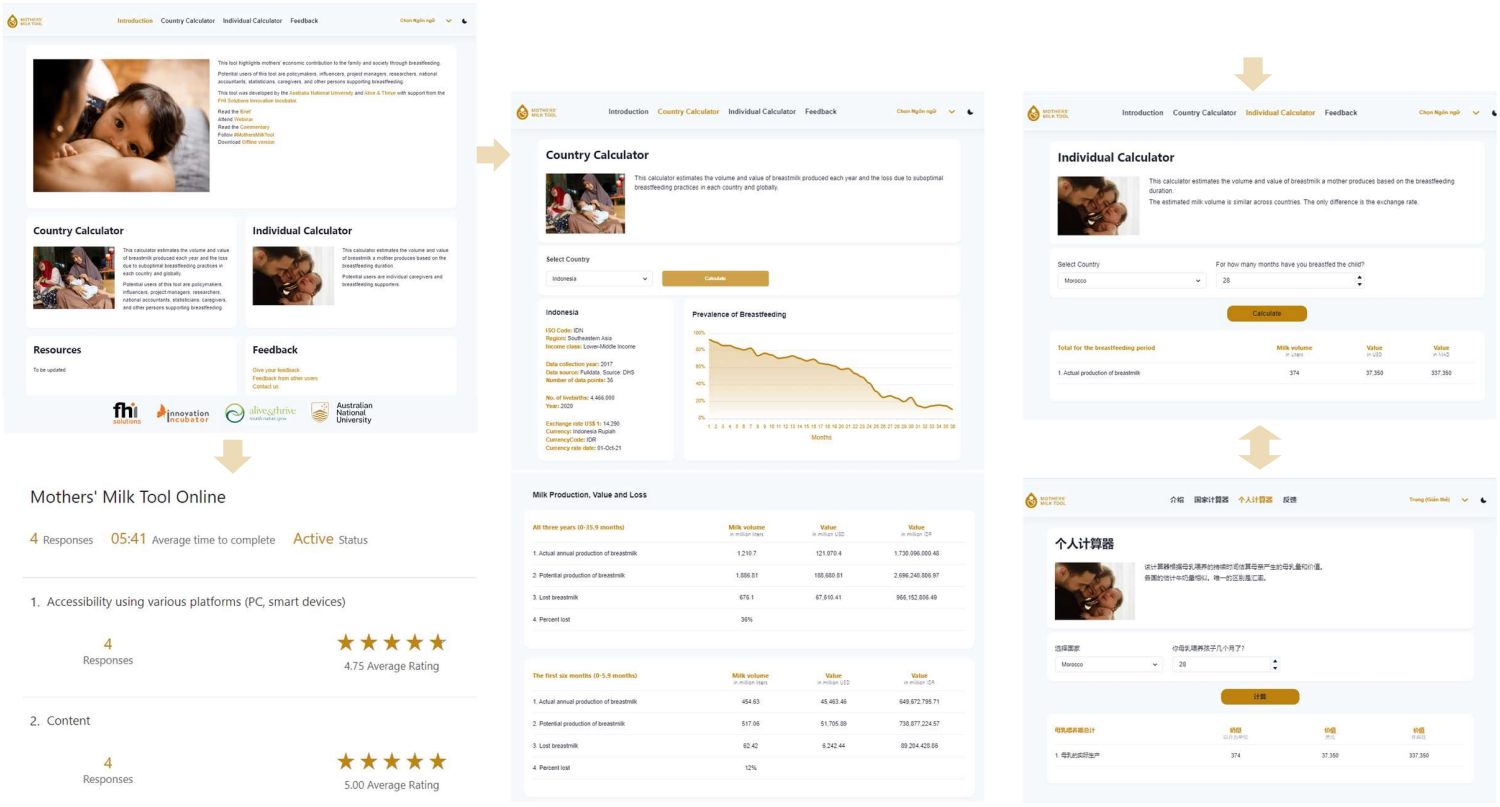
Layout and key features of the Mothers' Milk Tool online. Authors created this figure using snapshots of the Mothers' Milk Tool online (https://mothersmilktool.org). The human identifiable images are licensed for personal, business or commercial purposes.

After successfully launching the Mothers' Milk Tool offline, we developed the Mothers' Milk Tool online (Figure 2). We are collecting user feedback to continue improving both online and offline versions. The offline tool is available in English and French, while the online tool is available in almost all languages. There are challenges to the development and use of the tools. Breastfeeding indicators are not or only partially available or out-of-date in select countries, which alters the calculation. Countries need to collect and publish this data regularly. We need to use regional estimates or fill in the information using the predicted model. We need to search for newly available data to update the tool. The currency exchange rate and the number of children born each year have not been updated since the development of the tool. We plan to update the offline tool periodically and develop an option for updating background information in real-time for the online tool.

### 2.6. Reflexivity statement

This paper is written because of the researchers' shared beliefs that women's unpaid work including breastfeeding is not well addressed by conventional economic studies which focus on the market economy, to the disadvantage of women and children, and that this reduces the resources invested in programs which are important to the health of women and children in particular breastfeeding. Our focus is on low- and middle-income countries but our study includes global and high-income country perspectives due to our concern to highlight that the latter present a pathway on infant and young child feeding which may harm women's and children's health if followed by LMICs.

The authors include one female who is the lead author and three males, and the research team is based in Australia, Vietnam, and London. The three male authors have many years of experience in low- and middle-income countries on programs supporting maternal and child nutrition including in emergencies. The lead author is a former government economist and tax analyst and a qualified breastfeeding counselor in Australia with extensive experience and commitment to supporting women to overcome societal and personal barriers to breastfeeding and to advocate for societal changes to enable them to breastfeed to the extent they see as optimal for their health and wellbeing. The four of us have collaborated since 2020, based on a common interest in improving the measurement of the economic value of breastfeeding and the economic and health system costs of not breastfeeding. In this collaboration, we have sought to develop a robust tool in collaboration with the diverse users, which draws on positivist economic approaches to monetary valuation of non-marketed production yet is also respectful that many women and cultures view it as unnecessary and even devaluing to place a monetary value on breastfeeding. We also respect the loving care that mothers offer their infants and young children regardless of how they decide to feed their children in the circumstance of their individual lives.

## 3. Results

### 3.1. Global estimates and estimates for selected countries

#### 3.1.1. Global production

Global production was around 35.6 billion liters a year. This represents just under half the potential production if women and children 0–35.9 months were universally enabled to breastfeed optimally (Table 4). Valuing the lost milk at around US$ 100 a liter represents a monetary loss of production of US$ 2.2 trillion annually.

**Table 4:** Estimated amounts and values of actual and potential human milk production by country for children aged 0–36 months.

**Country/Location**	**Year**	**Total production, at current breastfeeding rates (million Liters)**	**Potential production of breastfeeding (million Liters)**	**% of breastmilk lost**
Australia	2010	50.8	143.2	64.5
Brazil	2019	425.4	1,212.9	64.9
Canada	2009	54.5	169.8	67.9
India	2017	8,737.6	10,200.0	14.3
Indonesia	2017	1,210.7	1,886.8	35.8
Ireland	2013	4.4	24.1	81.7
Kenya	2014	450.9	599.1	24.7
Nepal	2019	221.3	230.3	3.9
Nigeria	2018	2,150.4	2,997.1	28.3
Norway	2018–2019	10.7	25.3	57.8
Philippines	2017	574.5	826.0	30.4
United Kingdom	2011	58.0	314.3	81.6
USA	2018	604.5	1,686.1	64.1
Viet Nam	2013–2014	423.3	672.6	37.1
Global	2022	35,556.0	57,490.5	38.2

“Year” refers to the year in which available breastfeeding data is reported.

Key results for the selected countries (Australia, Brazil, Canada, India, Indonesia, Ireland, Kenya, Nepal, Nigeria, Norway, Philippines, United Kingdom, USA, and Viet Nam) and the world are presented in Table 4.

Among high-income countries, human milk production ranges from around 11 million liters in Norway, 605 million in the USA, and 51 million in Australia (countries where around two-thirds of potential production is lost) to 4 million liters in Ireland. In Ireland, around 80% of mothers' milk is lost.

Among low-income countries, Nepal maintains human milk production at high levels (221 million liters annually) with less than 5% lost. Other countries such as Kenya, Nigeria, and Vietnam currently lose around a third or less of production. Likewise, middle-income countries such as Indonesia and The Philippines lost around a third of potential production.

The most populous country, India, lost nearly 40%, respectively, with a production of around 8.7 billion liters a year.

#### 3.1.2. Monetary values of mothers' milk production

In monetary terms, the value of human milk production is substantial in most of the selected countries (Table 5). The monetary value of lost mother milk ranges from around US$ 146.2 billion in India to US$ 900 million in Nepal.

**Table 5:** Estimated production values and “lost milk” by country.

**Country/Location**	**Year**	**Value of total breastmilk production (million US$)**	**Value of breastmilk lost (million US$)**	**Predicted**
Australia	2010	5,079.55	9,242.6	Yes
Brazil	2019	42,538.66	78,756.1	Yes
Canada	2009	5,452.83	11,531.0	Yes
India	2017	873,755.44	146,244.7	No
Indonesia	2017	121,070.40	67,610.4	No
Ireland	2013	440.78	1,967.4	Yes
Kenya	2014	45,093.29	14,814.8	Yes
Nepal	2019	22,125.00	900.3	No
Nigeria	2018	215,038.69	84,670.6	No
Norway	2018–2019	1,069.53	1,465.4	Yes
Philippines	2017	57,446.25	25,149.1	No
United Kingdom	2011	5,796.39	25,636.3	Yes
USA	2018	60,451.21	108,161.7	Yes
Viet Nam	2013–2014	42,334.06	24,925.2	No
Global	2022	3,555,597.42	2,193,451.7	Yes

“Year” refers to the year in which available breastfeeding data is reported.

## 4. Discussion and implications

### 4.1. Key findings and strengths of the study

Human milk produced for infants and young children by breastfeeding mothers is a crucial national food system; this production contributes substantially to national and global food security and health, though much is also “lost”.

The economic value of this food production by breastfeeding mothers can and should be measured, to ensure that this important economic contribution is visible, properly valued, well-protected, and sufficiently resourced to continue.

A culture of breastfeeding is an important national capital asset with large economic value, which generates a substantial quantity of safe, nutritious, healthy, and environmentally sustainable food for a country's infants and young children. A supportive breastfeeding culture protects the reproductive health of women and minimizes food system pressures on the environment.

Where a breastfeeding culture is not visible, valued, and resourced, breastfeeding will diminish, and milk production capacity is lost, due to market pressures from commercial milk formula, hence countries' important 'cultural capital' of women's breastfeeding knowledge, skills, and experience should be protected, and investments made in breastfeeding protection, support and promotion to prevent and restore Lost Milk.

### 4.2. Limitations

The accuracy and capabilities of the Mothers' Milk Tool remain limited by the gaps in available data. The tool does not adjust for exclusive breastfeeding rates during the first 6 months because of data limitations for breastfeeding prevalence and milk intake. Breastfeeding prevalence data is particularly lacking in high-income countries. Up-to-date scientific knowledge is also lacking regarding the biologically feasible potential levels of breastfeeding and the usual human milk intake, particularly among young children.

Several enhancements have been identified during development that can be considered for future improvement of the tool. These include modifications to increase its accuracy, flexibility, functionality, and add-on modules to broaden the tool's capabilities.

For example, the basic model could be modified to recognize that infant and young child mortality is high in some countries, and the number of births will be higher than the number of breastfeeding children. Especially if better scientific data were available, greater flexibility could also be added to the tool to vary its assumptions about the milk intake of young children who are breastfeeding. Also, breastfeeding has some energy costs for the mother; users could be given the option of adjusting the monetary value of production for the cost of any additional necessary nutrition for mothers.

Modifications to allow other approaches to placing a monetary value on human milk can also be considered. Options include allowing the user to enter information on wages for women employed as wetnurses to calculate monetary values per liter of milk or per day of breastfeeding. Similarly, the value of maternal time invested in breastfeeding can also provide an input-based proxy for the monetary value of the milk produced. Estimates of maternal time inputs over the breastfeeding period could be incorporated into the existing tool using available data from time-use studies of breastfeeding and childcare. As commercial trade in human milk expands, using new sources of market data can also be explored for monetary valuation.

The individual mother component of the tool could be modified to provide production data for multiple children, and for distinguishing between months of exclusive and partial breastfeeding. Important but more complex programming enhancements that could be added to the tool functionalities for countries include per capita production estimates which would improve its value for cross-country comparisons, as well as flexibility and pre-loaded data to allow time trend analysis. This would also further assist in tracking progress against policy targets.

Where countries have policy targets for breastfeeding, the tool could be enhanced to measure the gap between the actual and target level of human milk production. The tool could also provide a page with a prefilled advocacy brief for explaining and presenting country results to policymakers in a suitable format to motivate and guide policy action.

Furthermore, by linking the estimates of lost milk production to country data on the volume of milk formula sales or usage, the Mothers' Milk Tool could provide a suitable platform for calculating environmental savings at current breastfeeding rates, and the potential costs (such as increased greenhouse gas emissions, and water use) of further declines in breastfeeding.

The scope for linking the Cost of Not Breastfeeding Tool to Mothers' Milk Tool results for lost milk production could also be explored. Together these tools can help present the investment case for breastfeeding. Furthermore, tools such as the WBC*i* Costing tool are available to estimate the financing costs of breastfeeding policies and programs (19). We suggest the need to also develop ways of linking these tools to facilitate formal economic evaluations of country-level interventions.

### 4.3. Policy implications

The tool provides the potential for many countries to revisit their current maternal, newborn and child health, early childhood nutrition, and food security strategies. Policymakers will be able to compare the large monetary value of these current losses against larger potential losses if current levels of breastfeeding are not protected; the ability to minimize losses by increasing social investments in building a more enabling environment for breastfeeding will also be made visible.

The tool can also illustrate the extent to which a country's breastfeeding practices are providing mitigation, adaptation, and resilience to climate change risks, and may assist with planning for humanitarian emergency responses.

Human milk is valuable for its nutritional and immunological characteristics. Using a market price to place a monetary value on it is possible because breastfeeding is increasingly commodified. Human milk and human milk products are being bought and sold. This raises important policy issues but is beyond the scope of this study. This important discussion of feminized poverty and lack of adequate policy support for breastfeeding as a key driver of commodification trends is considered elsewhere (52–54).

Several studies have looked at the cost of key policies to better enable women to breastfeed, though a recent review of costing studies concluded that the availability of cost estimates was limited and more standardized costing frameworks are needed (55).

### 4.4. Implications at the country level

The tool has been verified through comparison with published estimates of human milk production in several countries. This shows good alignment with estimations for a variety of settings and diverse methodologies.

The results show that the $6 billion daily value of lost mother's milk production can be considered alongside the US$1 billion a day of health and human costs directly attributable to not breastfeeding that has been calculated by the Cost of Not Breastfeeding Tool (2).

With advances in the state of scientific knowledge about the acute and chronic disease impacts of not breastfeeding, it could be expected that these estimates would increasingly align (44). For example, some health services are already willing to pay high prices for donor human milk as the health cost savings are well documented for premature or vulnerable infants, and this is reflected in the monetary values used in the Mothers' Milk Tool. However, there remain considerable gaps in data and knowledge about the broader maternal and child health impacts of not breastfeeding and the economic cost consequences. As evidence accumulates on the health differential for non-breastfeeding mothers and children, including for chronic diseases, the measured costs of not breastfeeding will tend to rise.

The lack of key data especially in high-income countries means the important trends in potential health costs and losses arising from insufficient breastfeeding are invisible to policymakers. There is an urgent need for regular, comprehensive, and accurate measurement of breastfeeding prevalence in all countries to track trends and inform a range of public policies. Systematic data collection on prices charged by human milk banks for fresh and pasteurized milk should also be prioritized and published regularly.

## 5. Conclusions

The Mother's Milk Tool estimates the volume of human milk currently being produced, and the volume that is potentially at risk if women's important production capacity for breastfeeding is not protected, promoted and supported by effective national policies and programs. It also calculates how much is currently being lost at national, regional, and global levels. Monetary values are also indicated.

The estimates show the breastfeeding mothers' substantial contributions to food production, and how much of this healthy and sustainable foundation food is lost or at risk as ultra-processed commercial baby foods, including conventional cows' milk-based commercial milk formula products, are more widely marketed. In some North American and European countries, as much as 70–80% of potential milk production is lost, a phenomenon arising from cultural barriers or structural impediments to breastfeeding. Some middle-income countries are approaching these levels.

The tool also informs on a range of other economically relevant consequences such as a country's potential educational attainments, human capital development, poverty alleviation, non-communicable disease prevalence, and policies for climate change risk, adaptivity, and resilience.

We anticipate the Mother's Milk Tool to be a user-friendly resource that is open-source, adaptable, and useful for a variety of users. The Mothers' Milk Tool can be used by policymakers, advocates, and researchers for their decision-making and programming, and advocacy on the seven policy actions set out in the 2015 Call to Action by the Global Breastfeeding Collective. The tool will especially support the tracking of progress on breastfeeding targets, by assisting food and health policymakers and national statisticians to include breastfeeding in food balance sheets and economic statistics.

This tool can also be used by individual mother/baby dyads to estimate the economic significance of their breastfeeding practices. Future development could include real-time currency conversions, languages other than English, and comparisons across countries, as well as provide for regular maintenance and improvement of the Mothers' Milk Tool.

## Data availability statement

The datasets presented in this study can be found in online repositories. The names of the repository/repositories and accession number(s) can be found in the article/Supplementary material.

## Ethics statement

Written informed consent was obtained from the individual(s) for the publication of any identifiable images or data included in this article.

## Author contributions

Conception or design of the work and critical revision of the article: JS, AI, TN, and RM. Data collection: JS and AI. Data analysis and interpretation and drafting the article: JS, AI, and TN. All authors have read and agreed to the published version of the manuscript.
