# Supplementary material for: The volume and monetary value of human milk produced by the world's breastfeeding mothers: Results from a new tool
Source: Front Public Health. 2023 Mar 30;11:1152659. doi: 10.3389/fpubh.2023.1152659 (PMC10098319; doi:10.3389/fpubh.2023.1152659)
Supplement: Supplementary file 1 [file Data_Sheet_1.docx]

Supplementary Material

The volume and monetary value of human milk produced by the world’s breastfeeding mothers: Results from a new tool

Julie P Smith, Ph.D.;^1,2 *^ Alessandro Iellamo, MPH;^3^ Tuan T. Nguyen, MD, MSc, Ph.D.;^4^ Roger Mathisen, MSc^4^

^1^ National Centre for Epidemiology and Population Health, College of Health and Medicine, the Australian National University, 62 Mills Road, Acton, Canberra, 2601, Australia

^2^ Crawford School of Public Policy, College of Asia and the Pacific, the Australian National University, 132 Lennox Crossing, Canberra, 2600, Australia

^3^ Independent consultant, London, UK.

^4^ Alive & Thrive East Asia Pacific, FHI 360 / FHI Solutions, 60 Ly Thai To Street, Hanoi, Vietnam.

* Correspondence: julie.smith@anu.edu.au

# Supplementary material 1. Key data sources and analysis behind the estimates.

1) Numbers of children aged 0-35.9 months

Estimates in the studies reviewed differed in how the number of infants and young children was measured. Most estimates used the approximate number of live births to measure the number of infants and young children of relevant age who were potentially breastfeeding during the relevant year. Few made any adjustment for mortality. In some countries, such as Norway, infant mortality is very low, and the effect of this approximation is not substantial. However, the review identified the need to allow for adjustment to account for infant and young child mortality for the relevant age categories in countries where this is high. A possible difference between the number of live births and the population of infants and young children may need to be considered in any cross-country or intertemporal comparisons. The tool does not adjust for infant mortality.

2) Breastfeeding rates available for the country of interest for specific age brackets (indicating exclusively breastfed, and partial or continued breastfeeding); assumptions about 2-5% cannot breastfeed; the prediction model was developed for calculating by month of age where there are data gaps. The tool uses continued breastfeeding rates for the age range 0-35.9 months.

Data on breastfeeding practices by month of age of infant/young child is the most appropriate input for the calculation and is used in many studies of the economic value of breastfeeding. However, many countries do not collect such data, or the scope of data collection does not include the full age range of 0-35.9 months. Data on exclusive breastfeeding also has many gaps. For many low- and middle-income countries (LMICs), the necessary data is more easily available, through periodic DHS or MIC surveys. However, it is not available for all LMICs.

We used the Demographic and Health Survey (DHS) (United States Aid (USAID) 2021) and Multiple Indicator Cluster Surveys (MICS) (UNICEF 2021b), which were carried out in 113 LMICs between 2010 and 2020, for which data is publicly available. These are nationally representative cross sectional household surveys which are accepted internationally as official data sources for various health and nutrition indicators for children and reproductive age (15-49 years) women. Both surveys use standard and comparable procedures across countries and are substantially consistent over time. The surveys (sampling, questionnaires, data collection methods, and validation procedures) are fully described. Both collect information on breastfeeding status of children born in the five years preceding the study, from interviewing the mother. The self-reported breastfeeding status of the mother at the time of the survey was ascertained through ‘are you currently breastfeeding in DHS (United States Aid (USAID) 2021) and ‘is the child still breastfeeding’ (MICS) (UNICEF 2021b).

In the case of high-income countries (HICs), data on breastfeeding prevalence is notoriously poor for many countries. A recent study reviewed data from HICs and confirmed the findings of the earlier studies (Vaz et al. 2021; Victora et al. 2016). Most HICs had limited data, and usually did not report breastfeeding rates past 12 months. Lack of data from HICs was also a barrier to estimations for the Cost of Not Breastfeeding Tool (Walters et al. 2019). The paucity of data is indicated by the following figure from the Global Health Observatory.


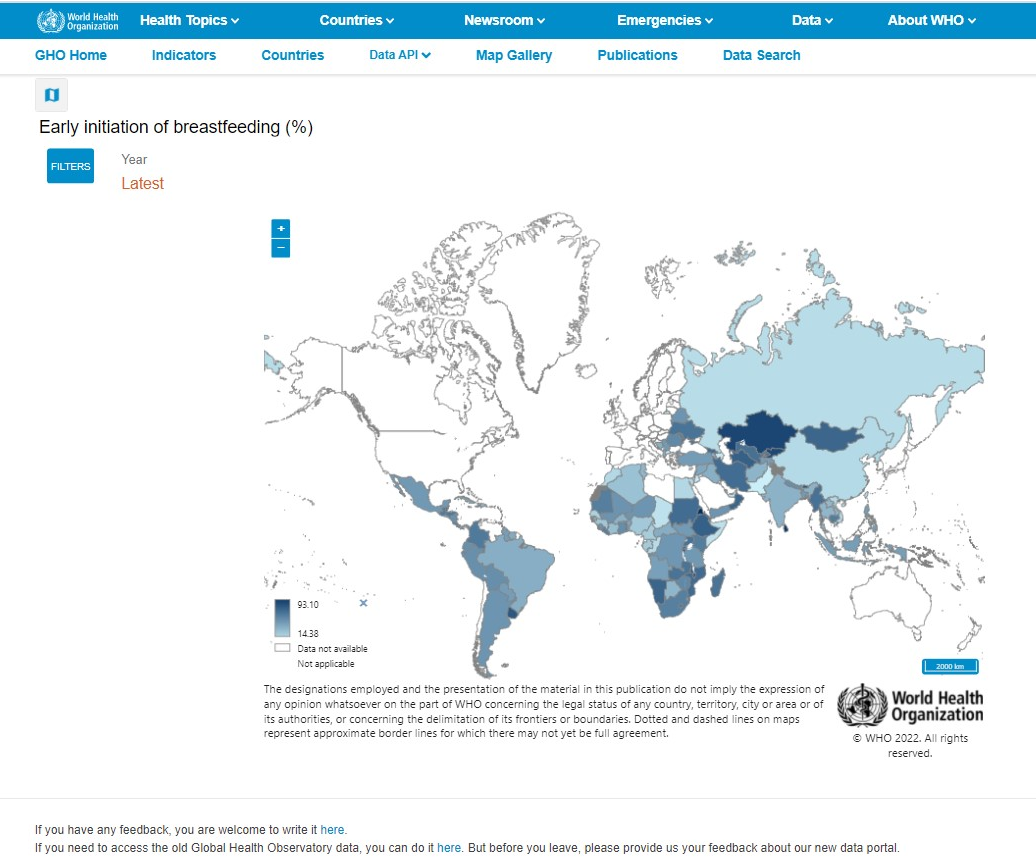

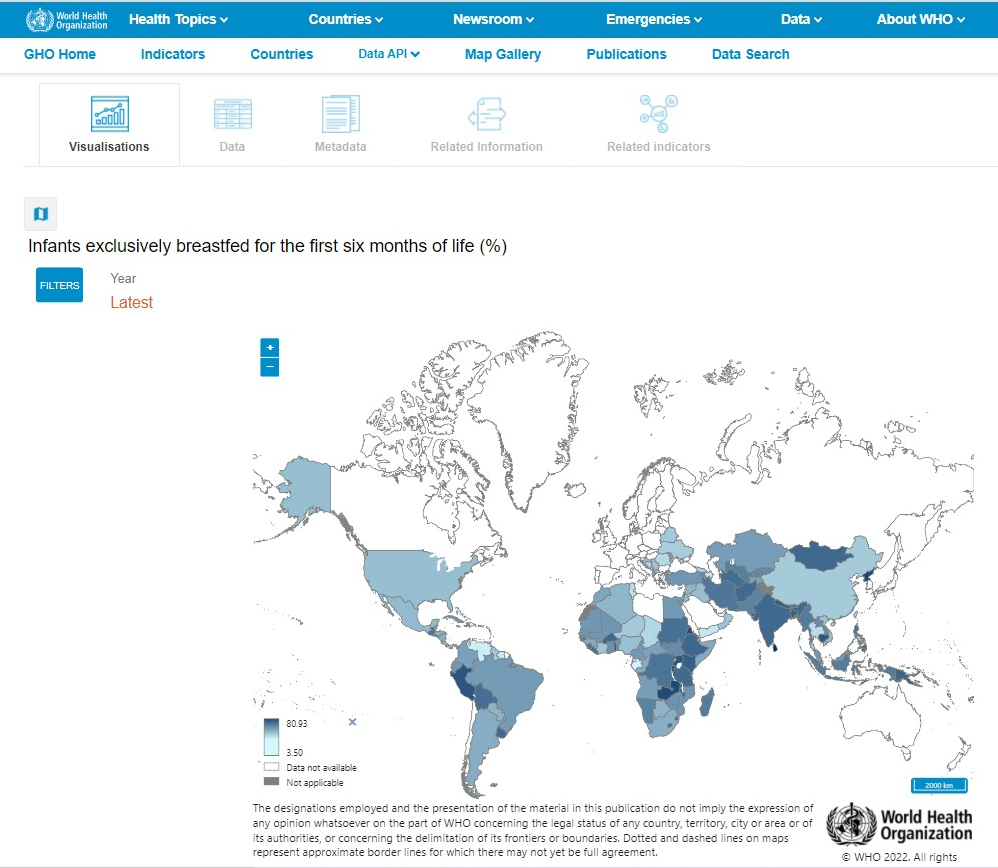


###### Figure 1 Global availability of breastfeeding data

In this study we developed a predictive model to fill gaps in country data for monthly rates of any breastfeeding, as follows. The model for breastfeeding rates was developed based firstly on review of studies of historical and cross-country breastfeeding rates, and then by testing of different statistical models at country and regional level.

The review of historical breastfeeding data was based on manual search and researcher knowledge of historical data sources. For example, long term historical breastfeeding data is available for countries such as Norway (Liestøl et al. 2008), Singapore, and the Philippines (Berg 1973), as well as the US (REFs) and some other European countries (Liestøl et al. 2008). Official historical data has also been compiled for some sub-jurisdictions, for example in Victoria, Australia (Mein Smith 1990). Norway and Australia were a particular focus, with data available back to at least the beginning of the 20^th^ century for both of these countries (Smith). An anthropological study in non-industrial populations also provided context and some quantitative data on traditional breastfeeding practices (Sellen 2001). We tested the tool for [18] countries using this historical data, by comparing results with published results from existing studies. To do this, historical births and breastfeeding data from relevant studies published since 1973 were the inputs into the tool, and the tool’s prediction model was used to fill gaps in breastfeeding data to calculate production volumes. As the milk intake of breastfed children is a fixed factor in the tool, the tool calculation also removed a source of inconsistency in estimates over time and across countries. This verification process also allowed identification of whether variance between published estimates and tool estimates was due to different methodologies or was mainly due to different assumptions about milk intake.

Secondly, data for selected years and countries was also tested using regression modelling to identify the line of best fit for the 0-35.9-month age group, using SPSS (version 27). This was tested for its accuracy after importing the estimated regression into Excel.

This analysis of historical and cross-country breastfeeding data at country and regional level suggests that the predictive model which best fits breastfeeding patterns for most countries is a non-linear (cubic) regression function. This gave an R^2^ of over 95-99% for most countries whereas the next best model, a linear regression function, gave good results of around 90-95% for most countries. This means that the function shows an upward concave function for the early months of breastfeeding and a downward sloping convex function during the weaning period, as illustrated in the case of Nepal in Figure 2 below. This was the case for both country level analysis and for analysis by region.


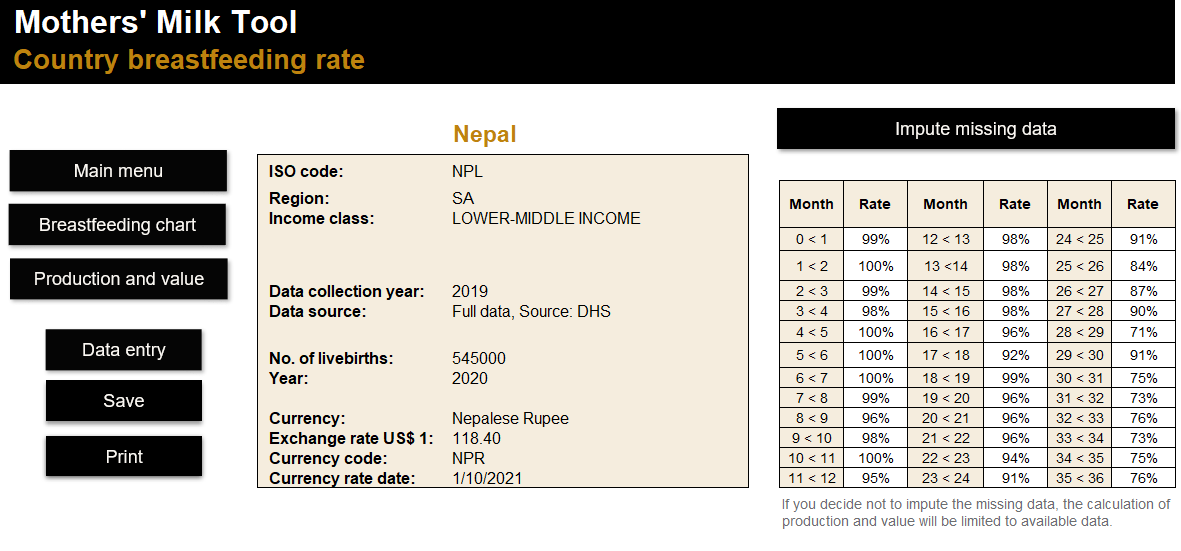


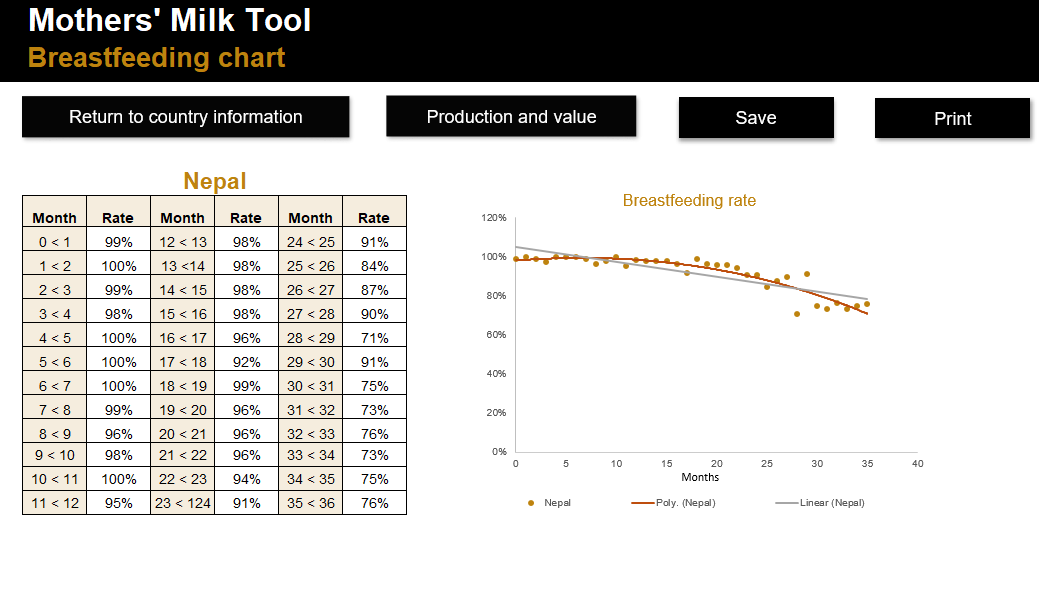

###### Figure 2 Actual breastfeeding rates by month for Nepal, 2019


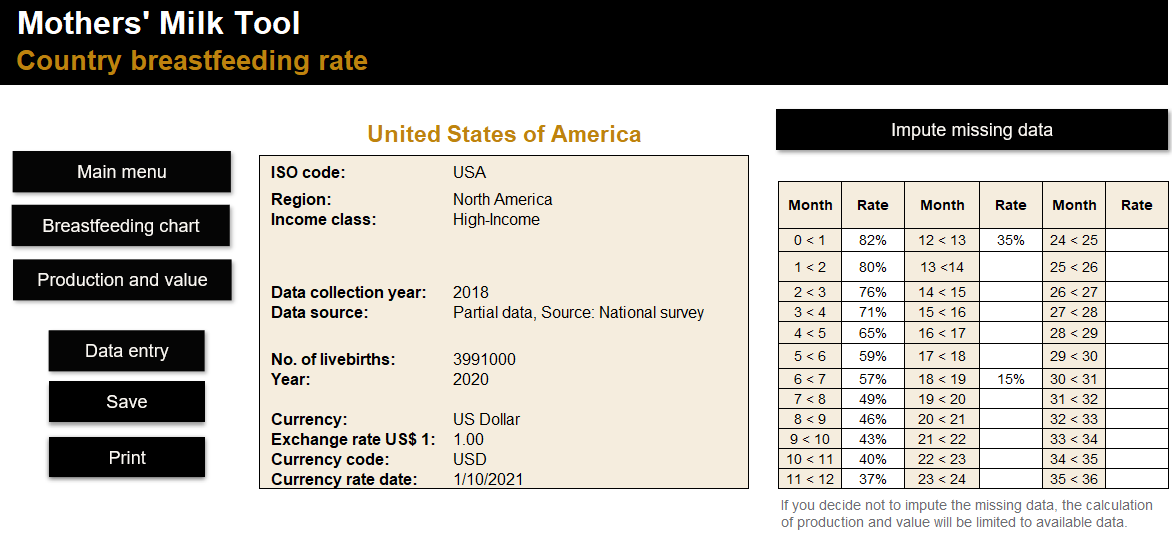

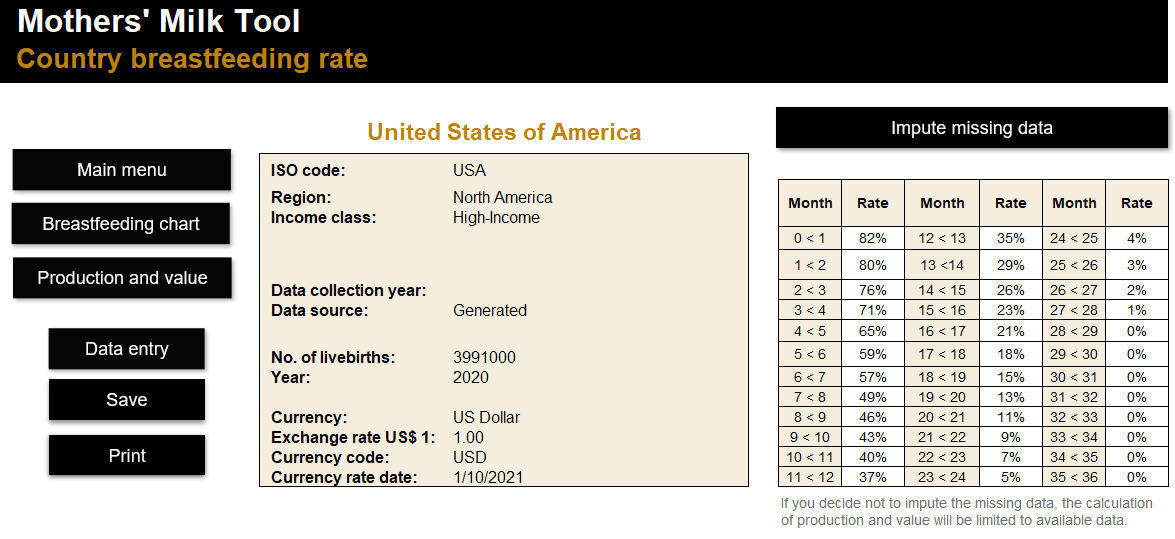


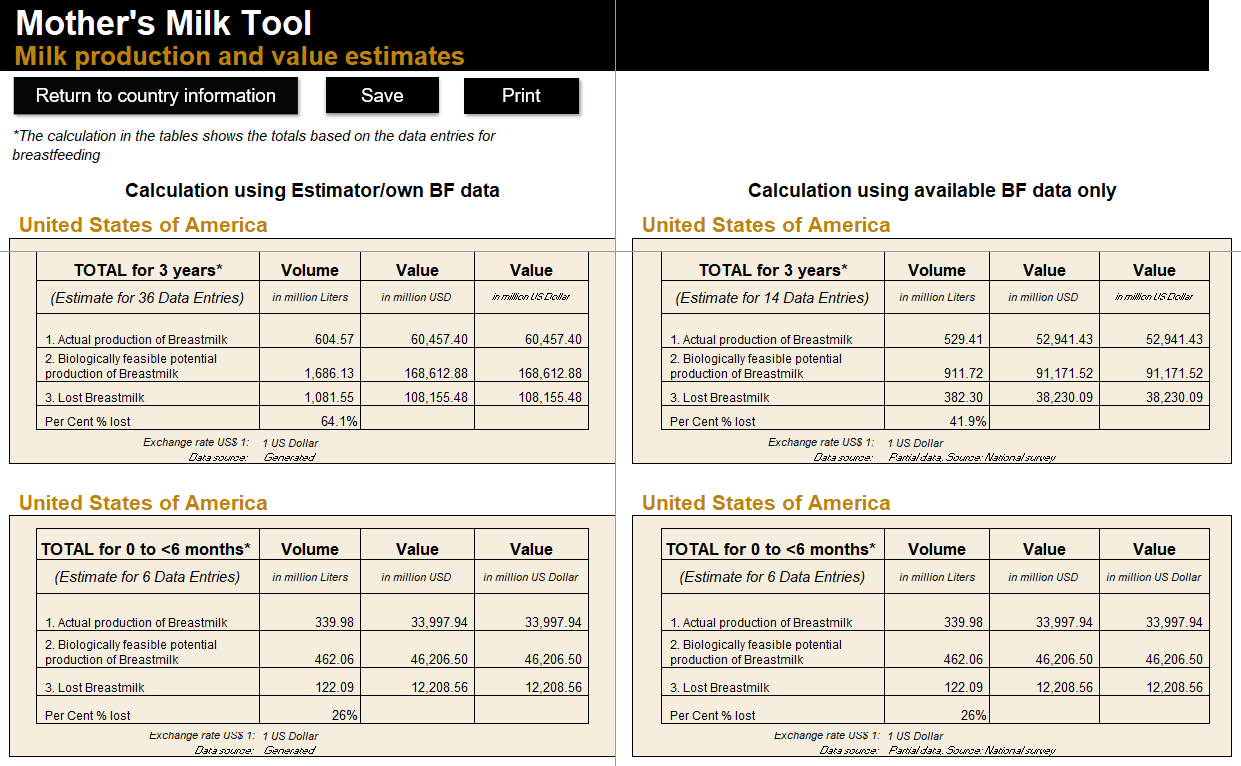


###### Figure 3 Actual and predicted breastfeeding rates by month for United States of America, 2018

That is, where there are gaps in rates of breastfeeding by month of infant-young child age, most of the variance in the prevalence of any breastfeeding for that month of age can be predicted by a correlational model using available data for breastfeeding by month of age.

To calculate the ‘lost milk’ when breastfeeding prevalence was below optimal/recommended levels, it was necessary to indicate the quantities of milk produced at the maximum biologically feasible levels of breastfeeding. To do this, the tool draws on a review of existing publications making such ‘lost milk’ estimates. Different authors used different approaches. For example, Berg takes a reportedly empirical position that 100% of new mothers are physically able to breastfeed their child for a period of at least two years.

3) Estimates of daily breastmilk intake by child age (day, months)

Studies used various assumptions about daily milk intake per child, depending in part on the extent of exclusive breastfeeding for the 0-5.9 month old population of infants. Evidence was also severely lacking on the intake for children beyond the first year. Rohde’s findings for Indonesia (Rohde 1974; Rohde 1982) as well as more recent studies which calculated milk production for children up to 24-36 months (Aguayo & Ross 2002b; Aguayo et al. 2001; Hatloy & Oshaug 1997; Ross et al. 2001) demonstrate that declines in breastfeeding prevalence for children in the second year of life and beyond are of considerable importance for early nutrition (Dewey 2001). The review identified the need for better evidence of milk intake, including in traditionally breastfeeding populations, to ascertain appropriate assumptions about biologically plausible milk production and milk intake levels for breastfeeding dyads. It may be necessary to update the tool assumptions about daily milk intake as new evidence becomes available. On the other hand, for monitoring trends, it is undesirable to make frequent or substantial changes to how production is calculated.

In some studies, the assumed daily intake implied overall intake during a 0-23.9 month lactation period ranged from around 290 (Rohde 1982) to around 310 (Smith 2017; Smith 2013; Smith 2018; Smith. 2012) liters. Many studies assumed much higher daily milk intake, ranging from an overall intake of 331 liters (Oshaug & Botten 1994) to as high as 443 liters (Aguayo et al. 2001) for the 0-23.9 month age range (Almroth et al. 1979; Berg 1973; Gupta & Rohde 1993; Hatloy & Oshaug 1997; Smith 1999; Smith & Ingham 2005; Smith et al. 1998). The assumption used in official estimates of human milk production in Norway is that the first 6 months of breastfeeding provides around 135 liters of milk and this is consistent with other studies (ranging from 108 liters (Rohde 1982) to 155 liters (Berg 1973).It aligns with a total of 306 liters for the lactation period of 0-23.9 months which provides the basis for official estimates of human milk production in Norway (Norway. Directorate of Health 2020). Also, the Norwegian assumption of 225 liters for the 0-11.9-month period is comparable with the range cited in other studies, of around 180-290 liters (Butte et al. 2002; Rohde 1982). On the other hand, its assumption of milk intake of around 80 liters for young children 13-23.9 months is conservative compared to other estimates, which range from 106 (Oshaug & Botten 1994) to 201 liters (Aguayo et al. 2001; World Health Organization (WHO) 1998). The Norwegian assumption about the human milk intake of this age group of breastfed children is considerably lower than even the lowest levels in other studies (Oshaug & Botten 1994; Rohde 1982).

Taking into account these considerations, the tool uses estimates of daily milk intake based on evidence collected by WHO for the first year,(Butte et al.) and by Hatloy and Oshaug’s 1997 review of studies on breastfeeding in the second and third year of life.(Hatloy & Oshaug) This provides for milk production of 211 liters for the first year, and 134 liters for the second year, for a total of 431 over three years of lactation. Data from these studies suggests that the best available estimate of milk production for the first six months is around 120-130 liters depending on exclusivity, and for the third year of milk production around 90.

Notably, the lack of data on daily milk intake, combined with gaps in data on breastfeeding rates by month of age, required the decision to base tool estimates on ‘any breastfeeding’ rates, rather than accounting for the extent of exclusive breastfeeding over the first 6 months.

4) A price at which to value human milk (such as price at which milk is sold or exchanged by human milk banks). There are prices of milk supplied by donor milk banks which exist in many countries of the world. These are typically not-for-profit operations, and most provide pasteurized human donor milk. In some countries, purchase of human milk can be arranged online via a website called Only the Breast. In some countries, wet nurses are employed, and by estimating the amount of milk produced by a wetnurse over a 24 hour period, a price per liter can be calculated (Smith). Key considerations in selecting an appropriate valuation method have been discussed elsewhere. For measuring the value of non-marketed production in economic statistics, there has been a preference for using the market price of an analogous product. It is also important that the price is reliably available, and desirably at the institutional level. Where milk is donated to milk banks by mothers who are not recompensed, the milk bank price will not fully reflect the extent of the value transfer. Market prices may reflect willingness to pay, but not ability to pay. Willingness to pay relies on full knowledge of the value of human milk, which means that well-informed purchasers and sellers are involved.

Considering these factors, it was preferred to use the price at which fresh human milk is exchanged between human milk banks in Norway to calculate monetary values of milk production. This price is currently around US$ 100 a liter. Donors in Norway are recompensed a small amount, and the price at which milk is exchanged is based on the institutional cost and updated by a regulatory body from time to time to reflect inflation. Information on the cost is documented. The value of human milk for children and health services is understood in Norway, thus, the price paid to milk suppliers may reflect a socially equitable rather than market-based, or ability-to-pay, distribution of the consumer surplus value. Other milk banks also provide prices but these are for pasteurized milk rather than fresh milk, and price data is less available from published or official sources. Likewise, prices for online milk purchases are from less reliable and consistent sources, reflect inaccurate prices, and reflect individual market participants rather than institutions.

#

# Supplementary material 2 on monetary valuation of milk production

| **Market** | **Price ($US per oz.)** | **Location** | **Comment/source** |  |
| --- | --- | --- | --- | --- |
| **Human milk banks** | | | | |
| ***HMBANA*** | $3-$4.5(Wikipedia 2013) | USA | Currently there are 12 HMBANA member milk banks providing donor human milk in the United States and Canada. HMBANA milk banks charge no fee for the actual milk, but charge a processing fee to offset the milk bank’s overhead costs. This fee ranges from US$3 to US$4.50 per ounce, plus shipping costs. Each milk bank has the authority to determine the processing fee for its facility, which is the reason for the wide variation in price. |  |
| ***Norwegian milk banks*** | $3.42 (US$100 per liter)(Grøvslien & Grønn 2009) | Norway | 13 milk banks were operating in Norway in 2009, all located in hospitals with level 111 NICUs. All preterm infants are offered donor milk if mothers' milk is unavailable or insufficient, and all infants who need milk from the milk bank are offered it. Donors are given a free hospital grade breast pump, and US$20 per liter to cover electricity and travel expenses, and donate for 6 months. At the main Oslo hospital where 2000 of the country’s 60,000 annual births occur, the milk bank collects around 1000-1100 liters of human milk per annum. There is a charge of US$100 for milk transferred to other hospitals. |  |
| **Viet Nam** | US$ 56 per liter (Mansen ; Tran) | Viet Nam | Currently there are 4 HMB in Viet Nam. The HMB received some external support (equipment, technical) for the establishment. The donors receive no compensation. Processing fees are collected to offset the milk bank’s overhead costs. This fee range of US$ 5-6 per liter is consistent across the network of HMB. Viet Nam has satellites of the HMB in nearby provinces. There is an additional cost for shipping of about US$ 5 per liter. The fee was estimated based on regulation of the government and approved by Provincial Department of Health. |  |
| **Internet milk exchange** | | | | |
| ***Only the Breast*** | US$1-$3 (Only The Breast 2013)  UK$2-8 (Only The Breast 2013) | Online | Milk can be bought and sold, as well as shared (donated). Exchange is organized into various categories, including by age of the infant, fresh (rather than shipped frozen), milk bank certified mother, milk bank screened mother, bulk sales, local sales, fat babies, special diet (vegan etc.). Site offers donor blood testing at US$219.45.  Also has trading from Canada, United Kingdom and elsewhere. |  |
| **Wet-nurse employment** | | | | |
| ***Wet-nursing*** | Daily rate between US$50 and US$200 (2012 prices).(Only The Breast 2013)  US$1,000/week (2007 prices) (Lee-St.John 2007) | USA | Offered at between US$50 and US$200 per day. Also has trading from Canada, United Kingdom and elsewhere. Equivalent to US$71-286 per liter at 700 ml daily intake. |  |
| ***Wet-nursing*** | US$2585/month (Fowler & Ye 2008) | China | Chinese wet nurses earned up to 18,000 Yuan/month in 2008. Exchange to US$ is based on 2008 exchange rates, and is equivalent to US$121 per liter at 700 ml daily intake |  |
|  |  |  |  |  |

# Supplementary material 3. on functional validation of Mothers’ Milk Tool

| Country/Location | Year of Publication | Age category | Total Mothers Milk Tool | Total Actual, Published | Variation (Actual-Published) | Variation (%) (calculator vs. published) | Reason for variance |
| --- | --- | --- | --- | --- | --- | --- | --- |
| Australia | 1992 | 24 | 30.5 | 33.2 | -2.8 | -9% | methodology to calculate, yields |
| Australia | 1992 | 24 | 30.5 | 33.2 | -2.8 | -9% | methodology to calculate, yields |
| Australia | 2013 | 24 | 40.2 | 42.0 | -1.8 | -4% | methodology to calculate, yields |
| Australia | 2012 | 24 | 42.6 | 40.3 | 2.3 | 5% | yields |
| Benin | 2002 | 24 | 6.2 | 8.5 | -2.3 | -37% | yields, considers BMS as a replacement |
| Bolivia | 2001 | 24 | 11.7 | 13 | -1.2 | -10% | yields, considers BMS as a replacement |
| Burkina Faso | 2002 | 24 | 13.1 | 17 | -3.8 | -29% | yields, considers BMS as a replacement |
| China | 2012 | 12 | 3703.2 | 3578.0 | 125.2 | 3% | yields |
| China | 2008-2012 | 24 | 1819.5 | 2344.0 | -524.5 | -29% | yields and population |
| Cote d'Avoire | 2002 | 24 | 13.1 | 18.0 | -4.9 | -37% | yields |
| Coutiala County | 1994-1995 | 24 | 0.12 | 115.8 | -115.7 | -93287% | methodology to calculate, yields |
| Coutiala County | 1994-1996 | 24 | 0.04 | 63.3 | -63.3 | -158150% | methodology to calculate, yields |
| Global | 2012 | 24 | 24953.1 | 23315.3 | 1637.8 | 7% | yields |
| Guinea | 2002 | 24 | 7.3 | 9.9 | -2.6 | -36% | yields, considers BMS as a replacement |
| India | 1999 | 24 | 4624.0 | 3944.0 | 680.0 | 15% | methodology to calculate, yields |
| India (URBAN) | 1993 | 24 | 1261.35 | 1209.0 | 52.3 | 4% | yields |
| India (RURAL) | 1993 | 24 | 3691.1 | 3202.0 | 489.1 | 13% | yields |
| India | 2012 | 24 | 6236.9 | 7003.0 | -766.1 | -12% | yields and population |
| India | 2005 | 6 | 1673.0 | 269.9 | 1403.1 | 84% | methodology |
| Indonesia (Urban) | 1982 | 24 | 850.19 | 582.0 | 268.2 | 32% | methodology |
| Indonesia (Rural) | 1982 | 24 | 1522.0 | 1052.0 | 470.0 | 31% | methodology |
| Mali | 2002 | 24 | 12.0 | 17.2 | -5.2 | -44% | yields |
| Niger | 2002 | 24 | 11.13 | 15.3 | -4.2 | -37% | yields, considers BMS as a replacement |
| Norway | 1992 | 24 | 7.4 | 9.5 | -2.1 | -29% | yields |
| Norway | 2011 | 24 | 10.0 | 10.5 | -0.5 | -5% | yields |
| Norway | 2018 | 24 | 9.5 | 10.0 | -0.5 | -5% | yields |
| Norway | 2005 | 24 | 9.9 | 10.3 | -0.4 | -4% | yields |
| Norway | 2020 | 24 | 9.8 | 10.1 | -0.2 | -2% | yields |
| Norway | 2013 | 24 | 10.0 | 10.5 | -0.5 | -5% | yields |
| Norway | 2012 | 24 | 10.3 | 10.4 | -0.1 | -1% | yields |
| Pennsylvania | 2007 | 6 | 1.95 | 0.4 | 1.6 | 82% | methodology |
| Philippines | 2012 | 24 | 534.4 | 466.9 | 67.5 | 13% | yields |
| Senegal | 2002 | 24 | 8.4 | 12.2 | -3.8 | -46% | yields, considers BMS as a replacement |
| Singapore | 1951 | 12 | 3.7 | 8.6 | -4.9 | -131% | methodology to calculate, yields |
| Singapore | 1960 | 12 | 2.4 | 6.0 | -3.7 | -154% | methodology to calculate, yields |
| Togo | 2002 | 24 | 6.1 | 6.2 | -0.1 | -2% | yields, considers BMS as a replacement |
| UK | 2012 | 8 | 51.6 | 46.8 | 4.8 | 9% | yields, considers BMS as a replacement |
| USA | 2012 | 14 | 485.5 | 525.1 | -39.6 | -8% | yields |
| USA | 2013 | 14 | 508.6 | 526.0 | -17.4 | -3% | yields |

NOTE: This table compares results from the original study, with calculations using the tool. The calculations using the tool use the same birth and breastfeeding data as the original studies, but not the milk intakes/yields assumed in those studies, so differences arise mainly from differences in methodologies or differences in assumed yields. Reasons for variance are indicated in the table on this basis.

# Supplementary material 4 on user validation of Mothers’ Milk Tool

| **General opinion on the Mothers’ Milk Tool and how it can contribute to overall breastfeeding advocacy** |
| --- |
| Please may I just give my first reaction after sitting down to use the tool. Just WOW!!! Honestly, my mind is blown!! And in truth I was moved to tears when I saw the numbers appear. Huge congratulations to all involved for such a huge amount of work! Seeing the concept presented with such sophistication and in a very usable format here is truly what the business world might like to call "disruptive". Having grown up on a dairy farm myself and seeing statistics like this so regularly shared from the Irish dairy sector it is such an historic moment to see this for mother's milk. I would go as far as to say it is a defining moment in the history of women in an economic context to see our hidden contribution presented in this way.  BUDGETS: In terms of advocacy straight off the bat here in Ireland I'm thinking of our current health Minister Stephen Donnelly who himself is an economist and that he needs to see this ahead with our pre-budgetary submissions to parliament asap, as well as our Ministers for Finance and Public Expenditure. In presenting a business case for investment an important piece is the ROI (Return On Investment) calculation for an investment over a period of time. It has made me think of for example our approximately 90% deficiency in no. of IBCLCs (Lactation Consultants) per live births according to published standards, and now with this tool we can illustrate the return to the country over time, never mind the additional costs incurred from the Cost of Not Breastfeeding tool.  Also thinking it's value for our National Breastfeeding Implementation Group in our health system and the assistance it will provide in underpinning their business case submissions for implementation of our National Breastfeeding Action Plan. We have huge gaps in our health system in meeting at least the maternity and community health components of an enabling environment for breastfeeding. I'm thinking already of all the relevant bodies where this could go to for example our national ESRI etc. to build on previous recommendations they have made to Government regarding investment in and protection of breastfeeding.  MORALE: In Civil Society orgs, in particular voluntary organizations the lifeblood of a movement is grounded in strong relationships and enthusiasm to provide energy to keep up advocacy efforts. I feel this tool will bring such a huge lift to the global advocacy movement for protection of infant feeding by seeing the value of the work in a new, tangible and compelling way.  I must admit I was very moved seeing the value of milk produced by the mothers of Ireland as well as the sadness of the lost milk, and no doubt the for milk that was never had and is often grieved for. Before even seeing my individual data I felt a sense of huge value in myself and all the mothers out there who might be contributing, without to date having any true recognition. Historically in fact many have been met with condemnation for daring the breastfeed.  The individual data is just sensational also. I'm so excited to let my husband know about that. I know the first question he will ask is ""And how was that calculated?"" :)  It would be great to have a little more background on how the value is assigned to human milk currently. Is it based on costs to transport/store etc.? Why such a high value? One irony that jumped out at me is that the total annual production if we had high rates would far exceed the annual production of cows milk which is so gloriously deified on this island: <https://www.statista.com/statistics/1276744/milk-production-value-ireland/>  I haven't done the math yet but I shudder to think how much we have lost in the decades past, at least in liters and how that has impacted on the health of our people. We have a couple of journalists here who have been making excellent progress and drilling into this information. There will be some sensational headlines to follow I expect and I can already see a couple of political champions who are emerging here that will be so empowered in our parliament chambers when speaking.  RISKS: My feeling is that this tool is so valuable it has the potential to be exploited by let's say, the forces of hyper-capitalism. Have you considered initially introducing an approval process before providing access to the tool. I'm thinking of the human milk processors/suppliers of the world and how that might provoke a commercial response. So initially just making it available to known organizations to be ahead of the game advocacy wise, before opening to the wider public. I suspect that particularly in Ireland we will hear a lot of "oh this kind of thing is shaming women even more, and just trying to guilt them into breastfeeding etc.,  Same kind of stuff we would hear in relation to the environmental stats for example. But we are well able to handle that now :) |
| Thank you, - it was fun using the tool! We believe it can be useful for training and in public health initiatives. |
| The tool will be an excellent asset for breastfeeding advocates to use when advocating for breastfeeding, especially when working with governments who are driven by economics. Some mothers, or groups of mothers, will enjoy using the tool to calculate their personal contribution |
| Great to help mothers see how they contribute to the GDP and economic value of their governments by breastfeeding, advocacy groups, advocating to governments to help them realize how they stand to lose out due to reduced breastfeeding |
| The tool is really interesting to have and to calculate food balance sheets assuming a political interest for it! |
| They are minor typos and reading ones. Good luck with the feedback and launch. A much needed tool and as I said before very impressive. |
| What an impressive and very much needed concept and tool. As I have been out of the workforce for over 10 years my comments below may simply be – me not being up to date with lingo/technology etc. |
| The tool looks very useful indeed. It will provide useful information on the value of milk production in countries (and the amount lost). From the national accounts’ perspective, this will provide important information for the analysis of (developments in) unpaid household activities. We are currently mainly focusing on time spent on specific productive activities, but this may put a value on the actual BF product provided. |
| For public use, it will need to have a really simplified interface like the “Cost of Not Breastfeeding” tool, which is a great way to generate lots of interesting and useful information. |
| I’ve given the tool a go - I’m not sure how much help I can be, as I’m no whiz at Excel. I find the online “Cost of Not Breastfeeding” tool from Organization 1 much easier to use - I assume that you intend to produce a similar online tool eventually? Overall, I LOVE this idea! |
| I think the tool will be very useful. Definitely useful for making economic arguments – submissions, political forums etc. |
| Love this tool! I think it is a very wonderful tool, making visible how much economic value is found in breastfeeding and human milk. It can be used for policy making and I am sure I will show this in the German National Breastfeeding Committee. We also have a new government with new chances of having influence (our minister of agricultural and consumer protection is now a “green” minister) and so this tool comes just in time. |
| It is an important tool, I believe the results (in monetary value) will be more interesting for policymakers. |
| Once I opened the tool, I can already see the hard work that was put into it. This tool is very important to measure the breastmilk produced by mothers and gauge the exact value. Since it has 2 tabs for COUNTRY DATA and INDIVIDUAL DATA, both the government policy making bodies and individual mothers can benefit because they can see the actual value of the breastmilk they produced. |
| The tool is amazing. I love it. It has the potential to be used in a multitude of ways. Just the other day, I was imagining how this might be used in divorce court, or social security benefits - it brings a very different element to valuing women's contributions to the household and national economy. |
| This is a great tool and it's important to make sure it's used widely, so it needs a good (funded if necessary) dissemination and advocacy plan to ensure that the tool is used and that everyone who works in IYCF considers it a reference point. "Many thanks for the opportunity to give feedback on the tool. Congratulations on a great accomplishment. |
| The tool looks really good and the instructions looked easy to follow but I couldn’t manage to open the tool after downloading as I got stuck in an endless loop of Microsoft telling me my password was incorrect, sending me a code, the code worked partially then stopped me going further, then I needed to get another code. So – sorry – in the end I didn’t manage to look at the full tool. Congratulations on this project. It’s going to be very valuable |
| Thank you for the updated tool. It now works very smoothly and it is very intuitive. I managed to get the data for the countries I looked at very easily, and the instructions were clear. I have no further ideas for improvement. Thank you very much for this tool and I will be glad to share this with my colleagues and especially with governmental institutions as soon as it is officially available. |
| "Well done to you both for your hard work. I think it is an excellent tool." |
| Well... congratulations for this brilliant job! |
| In Apple, main menu does not allow scroll down to choose country. When we continue on the Windows for India: We cannot make out what the Table on the right-side means (Impute Missing Data). Our understanding is that it is related to the Breastfeeding Rate shown in the graphic in the Tab: Breastfeeding Chart. (Source MICS 2017). In the Tab on production and Value: It surely makes sense that there is increasing loss from birth to 36 months. There we go to the Tables of 0-6 month for example how it was calculated as the main sheet shows 95% BF rates up to 6 months. Does that mean you only used the data on bf not on Ex Bf? It is hard to find the cost of human breast milk here; there is no regulation of such a price. We have just done an estimate from the net. on rates, we have done some data entry but it is for 24 months in the national survey. When we saved it into a new file (attached as Demo1) the sheet named “compare results” is coming blank (left side) |
| "We have tried using the tool and we think in the most parts it works very well and very informative. We have only several suggestions (attached), we hope this can help. Thank you very much for developing the tool and we look forward to the launching event, all the best! |
| "The tool looks very interesting, especially in country like XXX, where the policy makers including the development partners have never considered economic value of women's unpaid care for breastfeeding. I feel work the tool would be helpful for policy makers for advocacy and allocate more budget for breastfeeding, however, I feel it is challenging to translate the findings from the study into behavior in society like ours. I have provided my comments on the tool, but I feel sharing the methodology of the study on data collection process and duration would have been helpful to analyze the tool better. |
| This is such a great tool and we are looking forward for it to be launched publicly. |
| Very interesting to see the tool - obviously I went through "country XXXS"... first. |
| The tool is called ‘Lost Mothers Milk’ but it is more than the calculation of ‘lost’ milk i.e. milk that could have/should have been fed to an infant or young child. It is also about the ‘consumed’ milk and what that is worth (to the country’s economy) and that important fact is not captured in the tool’s title. |
| **Specific comments on tool** |
| The download didn’t work. |
| The excel file was a bit difficult to open. I tried to open it, but it said that it can’t connect to Organization 2 SharePoint (screenshot attached). After several time clicked OK button, it can open. For your information, I also opened using Organization 2 laptop. I don’t know how it looked on other computers. It might impact on some formula in the excel, as I saw there were some boxes with wrong formula (such as: #NAME? in several boxes). |
| We were not allowed to open the tool from our job e-mails at the Norwegian Directorate of Health and Norwegian Institute of Public Health, for security reasons. |
| "I downloaded the tool and then should give my Microsoft past word and was told that I have no access and cannot sign in! ""It should be that when you get such messages you just click close or X and it will then load up."" I had tried and this did not help to open :(""I am wondering whether it is if you are using an apple computer? Another person had problems with that … can you try on a different computer with a windows operating system")" I don’t have an apple computer I have a Microsoft PC! " "I have attached the web version here, after downloading it, clicking X X X X to close the message and then saving it. If you download this, save it to your computer and then open it as click on the X to get rid of the message, does that help?"" Works now!!! and I have even managed to enter the BF data |
| The excel file was a bit difficult to open. I tried to open it, but it said that it can’t connect to Organization 2 SharePoint (screenshot attached). After several time clicked OK button, it can open. For your information, I also opened using Organization 2 laptop. I don’t know how it looked on other computers. It might impact on some formula in the excel, as I saw there were some boxes with wrong formula (such as: #NAME? in several boxes). In the Main Menu sheet, I’d suggest to not using the arrow figure after “Select Country” box, as it confuse if it is a sequence (does it mean you have to click “Select for mother’s calculator” after clicking “select country” button. |
| I searched for Luxembourg and then there was wrong BF data already filled in, I did not press any button! Any use to send you the data to enter it in the system? For Luxembourg it gives strange BF statistics and no data source. The data source we have is already outdated and a new survey was planned but was blocked due to COVID so years will pass until having valid actual data! We don’t have data for all the end point listed in the table and this is the same for most of EU Countries, who only have irregular surveys not even with representative data. Does this still work? But I assume that the quality of generated data will not be that good in this case. Are you planning that policy makers enter their national data or do you link it to and international database with breastfeeding statistics? Interested groups of citizens might not be able to have the data to fill in! |
| The pdf instruction is easy to understand. |
| I don’t have any remarks about instructions or visuals. It is very intuitive. |
| Instruction are good that you emailed in a PDF. if that was accessible in the tool that would be great |
| it works great as it is. Instructions were clear and I found it easy to navigate my way around on a laptop. |
| On the Introduction PDF attached - a slight rewording of this might clarify it for some. Suggest emphasize/highlight the word ‘prompt’ - I had to read it several times before it sank in what it meant. Could be just my lack of skills !! |
| I found it easy to understand especially with the pdf instructions  You could also allow people to contribute to the amount of lost milk by also factoring in considering social equity that maybe including somehow that people also donate milk to milk banks, that this value and lost potential is also addition to the amount of breastfeeding. |
| I did not find the section on introduction and main menu very useful, rather a bit confusing. Would it be better to start directly with the country data estimate? |
| Instructions are fine – it’s just getting used to them! Visuals are also OK Explanation – the milk tool information sheet is good |
| For the ‘Mother calculator’, it is explained in the instruction that ‘you can estimate for multiple children only if they have been breastfed the same months and number of months. If not, then you need to estimate it individually’. Although this does make sense, it isn’t immediately clear from the excel sheet. I am wondering whether a sentence on this might be useful. Alternative is to provide people with multiple columns on the basis of number of children specified. This may be easier if you think that the months of breastfeeding might often differ between children. Furthermore, I noticed that it states ‘Then enter “Y” or “N” for each month the child was breastfed’, but guessing that Y is referring to ‘Yes’ and N to ‘No’, it should probably be rephrased a bit to explain that ‘Y’ has to be entered for each month the child was breastfed and ‘N’ for the other months’. It might also work if people only enter ‘Y’ for the relevant months and leave the rest empty. |
| In the ‘Milk production and value estimates’ sheets, information is provided on the ‘actual annual production of breastmilk’. I guess this estimate is based on the number of infant and young children aged 0-36 months; breastfeeding practices; and estimates of daily mothers’ milk intake by child age, as explained in the instructions, correct? And I guess this is what is referred to by ‘lost milk’, i.e. the value contributed to society by women’s unpaid care work through breastfeeding of infants and young children? However, the sheet then also contains information on ‘biologically feasible potential production of breastmilk’ and ‘lost breastmilk’. I don’t have the impression that these indicators are explicitly referred to in the instructions (or I may have misinterpreted some information) and I think they may lead to some confusion. In that regard, the reference to ‘lost’ breastmilk in the latter one, is different from the lost breastmilk in the generic sense of the tool, correct? Maybe I am missing something, but just wanted to check. |
| On the introduction page, I would remove cell F6 (referring to ‘LOST MILK TOOL’) as this is already captured at the top. Removing it might benefit the readability, as people might otherwise confuse that this also includes a link. |
| In the sheet ‘Mother calculator’ the cells H-P22-23 turn into an error when selecting a different country. It also seems that people need to fill out the price per liter in local currency. Isn’t there a default available, because I think quite a few people won’t have this available. Finally, in the results’ section, it wasn’t immediately clear to me what the six different tables represented. It may be useful to specify the time references (what seems to be the main difference) in the titles. You may also consider presenting it in an alternative way, showing the different time dimensions together under each category (although this might lead to a much bigger table, so it really depends on what users are looking for). |
| I noticed that if you click on ‘return to main menu’ in the ‘mother calculator sheet’, the two sheets ‘mother calculator’ and ‘MCalibration’ immediately disappear (and the sheet ‘country data’ reappears). This means that if people have not saved their results, this will be lost. It may be good to clearly highlight that, as some people may be caught by surprise by this. I also notice that this is the case for clicking on the ‘return to country information’ on the ‘result-lost milk estimate’ sheet and on the ‘BF rate chart’ sheet. |
| Re tools for mothers, we are not sure that is so useful. |
| For the ‘country data’ sheet, I see that after clicking on a country name, people have to check whether the table on the right is complete. If not, they need to push the ‘generate BF estimates’ button, but this was not immediately clear to me. It may be better to use a clear label for the table and then have a button that states ‘complete the table’. Alternative is to already complete the table upfront but specify which results are directly taken from the data source (specified on the left) and which ones have been estimated. Furthermore, on this sheet ‘years’ are included referring to ‘BF data year’ and (I guess) the ‘number of livebirths’ (this may need to be specified in the sheet). This made me wonder whether you would have some countries where you would have data available for multiple years and whether (in that case) people might select the specific data for the year they are interested in (or is it always showing the latest available year)? Particularly for people interested in time series analysis, this may be of interest. This may then also feed into graphs that show trends over time. |
| It seems that ‘references and literature’ and ‘definition of terms’ are now mainly linked to 2.1, whereas they may perhaps also relate to 2.2? In that case, you want to remove them from the specific dark grey area (and from 2.1 in the instructions) to more generic information. Otherwise, it might also be useful to add possible literature and definition of relevant terms for 2.2. |
| I am not sure how you plan to show the introduction and instruction in the excel file, but I think plain text (that people might read via a ‘fold out’ option) might work better than a comment field (that currently seems suggested). But maybe this was already your plan in the first place . |
| I am wondering how to read the difference between 2.1 and 2.2 (both in the instructions and in the tool). Do you also need to ‘select country’ if you want to run ‘mother’s calculator’? Because if that is the case, you may need to remove the ‘select country’ from the darker grey part of the tool which now specifically seems to refer to generating calculations of milk production and values, and present it as a generic issue, that needs to be done in any case. When using the tool, I see that the country selection can be done after clicking on ‘mother’s calculator’, but it may be useful to already ask for that selection at the start, to be in line with the approach for 2.1. The main menu also seems to imply that this is the recommended approach by stating the command in cell F7 (which seems to apply for both 2.1 and 2.2). Please note that it may in this case also be useful to change/add labels in the sheet (and instructions) to clearly distinguish between the two approaches (i.e. calculate national data versus calculate individual mother’s data). This may make the use of the tool more intuitive from my perspective. |
| I think the graph generator is very useful. The only thing that might be needed is labels for the axes and possibly a reference to the reference year in the title. I think also clearer labels may be needed for the series, e.g. the dots now have the country name but actually refer to the BF rate per month. Furthermore, it is not clear to me what ‘Poly.’ refers to in the second series and what the linear is referring to (how has this been determined and how should it be interpreted?). |
| Colors/font/sizing/buttons/drop downs etc. all very good, perhaps an automatic graphic generator like a simple pie chart or something like that might make it easier for more visual type people? |
| I like the simple old fashioned visuals |
| The instructions specify that the tool contains two tabs, but it already shows three from the start. You may need to reference this third one as well (or is it intended to be hidden? … in that regard I noticed that this specific sheet disappears when clicking on running the ‘mother’s calculator?).  Furthermore, more tabs may pop up dependent on what function is selected in the tool. It would be good to also briefly mention that at the start (and also explain that they might disappear if you return to the main menu (see later on)). |
| Mother calculator: very difficult to enter the number of children etc. (couldn’t enter directly into the box, had to click up on the cell)  Country chooser got stuck: Couldn’t choose any country further in the alphabet than Reunion  Was able to enter “y” for each month. Hard to interpret the charts: 36 “data entries” - is that 36 months? suggest you say “months” for mother-facing tool. The tool asks how many children, but the chart is just filled out once up to 36 months - how do you enter subsequent children?  How do you account for the percent “lost” when I entered all 36 months as “y”? I did not succeed in “saving” any of my work, but I did manage to print it out - however I was not given any choices about the format of printing, and the charts printed out off the edge of the page etc. so the printouts were not particularly usable, although it would be really useful if I could have printed them out all aligned etc." |
| “Application” failed and “BF rate chart” appeared as new sheet / tab at the bottom with a padlock - Same thing happened when I tried to save the chart. (the chart is very cute by the way with the graphics of the breastfeeding mum) |
| The chart could use more information in the legend - what is “Poly”? What is the X axis? |
| Recognizing that many of the issues I had are probably due to my poor Excel skills, here are my comments from my experience:  Opening the file, my computer warned me about macros and links to other websites - I accepted them all Then it told me it would open in “Read-Only” (I have Microsoft 365, Excel version 16.57) |
| Introduction: the “Introduction” and ‘Instruction” pop up boxes were blank. The other green boxes didn’t open to anything (so none of the definitions etc. would open for me). |
| Country: countries on list were tiny, even zoomed to 100%, and overlapped, so it was extremely difficult to click on the desired country. Moving the arrow down the list was incredibly slow. Where are the definitions of “breastfeeding rates”? I assume they should be available in the “Link to definitions” on the first page that I can’t open? Likewise the references? |
| Does the tool use exclusive breastfeeding rates? or “any” breastfeeding under 6 months? presumably after 6 months, it assumes “any” breastfeeding in addition to complementary foods? |
| I chose “UNITED KINGDOM” what is the source of the month by month data? when I clicked “Save” the chart of breastfeeding rates, I got this: Run-time error ‘1004’ Method ‘GetSaveAs Filename’ of object |
| The Excel spreadsheet is a simple, very plain format (visually unattractive), but it is adequate for the task and is a format that is available to most. |
| 2**. What might we improve**? |
| In the Main Menu sheet, I’d suggest to not using the arrow figure after “Select Country” box, as it confuse if it is a sequence (does it mean you have to click “Select for mother’s calculator” after clicking “select country” button." |
| Some labels could be further explained. I think it may also be useful if some generic text could be added in the sheets to explain a bit more how it could be read. |
| Overall the tool looks very simple and understandable from the first page. The use of picture is a good way to catch the attention of the users and read the details and explanation. a. Instructions? I suggest for the instructions, there would be and individual comment box when we hover over each tab to be sure what output/data is needed in that tab. COMMENT BOX will be helpful to easily think what is the correct info to be placed inside. |
| The emails clearly talks about the objective of the tool, nevertheless, a little more information on the methodology and who and how the data is collected would have been helpful to understand the tool better. |
| Any reason to focus on 3 years? We here in XXX focus on 2 years of children, and I think even the DHS survey only collects it for children under 2 years of age. In that case how are we planning to collect the information |
| I am not sure if the information provided in the individual calculator collates into the country calculator, if so why do we need data from DHS, if not, how is the information in the individual calculator going to be used |
| c.. Explanations? For non-English speaking countries, the explanations are very well understandable. It is not very complicated. |
| d. Others- There are some tabs in the tool that has the #NAME but I don't know what's that for. If we should put our name or not |
| Sheet Definition of variables: Line 13: we think the formula in column G can be clearer |
| Sheet BF Rate Chart, cell D21 it is supposed to be 23 < 24 |
| Sheet BF Rate Chart, any reason to put 0 to 36 months instead of 24 months? |
| We are making dummy numbers to try out the own data feature, but unfortunately, we cannot produce a result, as seen in the picture below: |
| On the sheet mother calculator, as Y represent a month of BF, what if the mother only BF for, for instance, 15 days? " |
| **a. Instructions** |
| The point “Impute missing data” needs more clarity and explanation |
| The instructions are pretty clear; however, can we explain that the individual calculator is of the individual child and the country calculator is for the country in the main instruction page. I was also not sure who enters the data for the individual sheet or how do we collect the data. Probably the webinar on the 5th May would be helpful to learn more. |
| I feel that it is not clear in the INTRODUCTION when you mention "the price of human milk". Better to explain. |
| **b. Visuals** |
| I love the visuals especially with the father in the picture. This shows that great immediate support comes from the spouse for their breastfeeding journey. |
| The visual to BF rate seems informative, However, can we also have any infographics for the cost of production of breastmilk, although I am not sure if that is possible? |
| **3. What are the things you would like to see/be added** |
| Explanations? It would be nice to see more explanation on the estimate/source for the value per liter of human milk. |
| Another suggestion: the replacement of breastmilk by formula milk has the price of formula milk - isn´t it? (at least 0-6 months for sure) - if this price has different costs in different countries how do you calculate, if you do not include it according to country? |
| In the country chart - don't you think it would be nice to include this price? and the person can make this comparison - human milk versus formula |
| I did not find any place to enter the time period for the information, it might be helpful to have that information in individual sheet as well |
| P.S. I was inquisitive to learn how did you calculate the cost of the breastmilk / liter? " |
| What is the price of BM the calculation is based on!" |
| I would like to see that the price of 1 liter of human milk in the currency of the country was put on the country sheet. I did have to look a while where to find it and it is the basis of the calculations. |
| Can we do something with an Exclusive Breastfeeding Rate for 0-6 months? |
| It was too difficult to add in the breastfeeding data for Aotearoa New Zealand – as I mentioned previously we only have at discharge from hospital data, 6 weeks, 3 months and 6 months data available. Our data is also collected as exclusive, fully or partial – or with exclusive and fully lumped together. We also do not have access to any data that is reported after 6 months so could not complete the one year box and certainly nothing past one year. |
| I am not sure whether you would need ‘references and literature’ and ‘definition of terms’ on the ‘main menu’ tab, when they are already available from the introduction (unless it would concern specific information per country). Generic reference and literature would from my perspective indeed make most sense on the introduction page. Definition of terms may indeed go under ‘main menu’, but I would then use a different color, to differentiate from the buttons to run specific calculations. |
| Perhaps an FAQs document with Qs such as ""How can I use the data in this document as a Civil  the tool for individual mothers to calculate their own stats doesn't allow them to have different feeding practices for each infant, could it be that they could specify what the practices were for each different child? |
| I think cross-country comparisons would be useful. Not sure how easy that is to do, but for example, in the chart, the country result could be compared with the average of countries in the same region or countries worldwide. I am also wondering whether some time series analysis might be possible, but this will really depend on the available data. |
| It would be helpful to have the complete reference (and URL) for the data source in the Country Information. This will be useful for people who don’t know all the sources of breastfeeding statistics and also to ensure that the most up-to-date information is being used, in case the tool isn’t being updated. |
| The figures on total volume of milk e.g. for 0 < 6 mo., the first year and the second year, are not the same as in our submitted paper on milk production in Norway. Could the reason be different estimated volumes per months than we have used in our paper? |
| Typo ….. PS: P.s. Note you can change any of the information |
| I’d suggest to give explanation on how to interpret the results. |
| I would like to see included the "how to do" more than only the indicators or variables. Maybe an attachment with a "formula" - I know we should go to your references and find the "way to do", the calculations. But... why not to simplify and include? |
| Overall I think the tool is already ok, except for the comment box when we hover over the tabs |
| This needs to be something quick and easy to pull up on your phone in a meeting with MoH, or to print to add to a report. I don't yet have a sense of whether it's that easy. |
| I’d also suggest to make a video instructions, in addiction to pdf file, showing how to fill in the excel. Make the video using Zoom recording is sufficient. |
| Sheet Definition of variables: Would it be possible to add an extra column after the formula of the measurement used? |

| Sheet Definition of variables: Line 26 – 30  need more information on BM yields (what does it mean in simple language) so users can differentiate between BM yields and daily yields per child |
| --- |
| Sheet BF Rate Chart, we fell we need more description regarding the % presented, is it cumulative? If not, perhaps cumulative table is also required |
| **4. What are the things you don’t think are necessary?** |
| I don't see anything extraneous. I think order is important for readability/useability so make sure the main menu comes up before definition of variables or that the main menu button is bigger. |
| None |
